# Galidesivir Triphosphate Promotes Stalling of Dengue-2 Virus Polymerase Immediately Prior to Incorporation

**DOI:** 10.1021/acsinfecdis.3c00311

**Published:** 2023-07-24

**Authors:** Sandesh Deshpande, Wenjuan Huo, Rinu Shrestha, Kevin Sparrow, James M. Wood, Gary B. Evans, Lawrence D. Harris, Richard L. Kingston, Esther M. M. Bulloch

**Affiliations:** † School of Biological Sciences, University of Auckland, Auckland 1010, New Zealand; ‡ Ferrier Research Institute, Victoria University of Wellington, 69 Gracefield Rd, Lower Hutt 5010, New Zealand; § Maurice Wilkins Centre for Molecular Biodiscovery, University of Auckland, Auckland 1010, New Zealand

**Keywords:** RNA-dependent RNA polymerase, Flavivirus, nucleoside analog, antiviral, SYTO 9

## Abstract

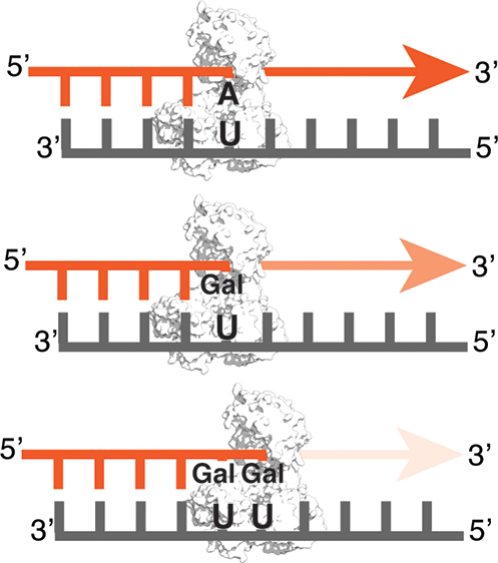

Millions of people are infected by the dengue and Zika viruses each year, resulting in significant morbidity and mortality. Galidesivir is an adenosine nucleoside analog that can attenuate flavivirus replication in cell-based assays and animal models of infection. Galidesivir is converted to the triphosphorylated form by host kinases and subsequently incorporated into viral RNA by viral RNA polymerases. This has been proposed to lead to the delayed termination of RNA synthesis. Here, we report direct in vitro testing of the effects of Galidesivir triphosphate on dengue-2 and Zika virus polymerase activity. Galidesivir triphosphate was chemically synthesized, and inhibition of RNA synthesis followed using a dinucleotide-primed assay with a homopolymeric poly(U) template. Galidesivir triphosphate was equipotent against dengue-2 and Zika polymerases, with IC_50_ values of 42 ± 12 μM and 47 ± 5 μM, respectively, at an ATP concentration of 20 μM. RNA primer extension assays show that the dengue-2 polymerase stalls while attempting to add a Galidesivir nucleotide to the nascent RNA chain, evidenced by the accumulation of RNA products truncated immediately upstream of Galidesivir incorporation sites. Nevertheless, Galidesivir is incorporated at isolated sites with low efficiency, leading to the subsequent synthesis of full-length RNA with no evidence of delayed chain termination. The incorporation of Galidesivir at consecutive sites is strongly disfavored, highlighting the potential for modulation of inhibitory effects of nucleoside analogs by the template sequence. Our results suggest that attenuation of dengue replication by Galidesivir may not derive from the early termination of RNA synthesis following Galidesivir incorporation.

Flaviviruses are significant human pathogens whose impacts are expanding due to both anthropogenic climate change and the increased mobility of human populations.^1,2^ Flaviviruses (Family *Flaviviridae*, Genus *Flavivirus*) are positive-sense, enveloped RNA viruses, which are usually spread between vertebrate hosts by arthropod vectors, such as mosquitos and ticks, and are most prevalent in tropical and subtropical regions. Important endemic and emerging flaviviruses include dengue virus (DENV, serotypes 1–4), Japanese encephalitis virus (JEV), Tick-borne encephalitis virus (TBEV), West Nile virus (WNV), and Zika virus (ZIKV). When humans are infected with a flavivirus, the resulting disease is sometimes mild, but potentially fatal hemorrhagic fever and neurological disorders can also result. In addition, unique clinical manifestations have been reported, such as fetal microcephaly caused by Zika virus.^3^

Vaccines are currently available for JEV, TBEV, YFV, and DENV, and clinical trials are being undertaken for ZIKV and WNV vaccines.^4,5^ However, existing flavivirus vaccines are of variable efficacy, and their development has not always been straightforward because of the complex immunological response to infection by different flaviviruses, and the presence of multiple endemic flaviviruses in tropical regions.^6,7^ Despite more than 60 years of research, there are currently no FDA-approved antivirals to treat flavivirus infection, and there is an urgent need for these to be developed.^8^

One widely utilized therapeutic approach targets the central process in all viral replication cycles by using nucleoside analogs to inhibit virally directed nucleic acid synthesis.^9−11^ In this study, we investigate the inhibitory effects of Galidesivir triphosphate (Figure 1), an adenosine triphosphate analog with broad-spectrum activity against RNA viruses, including flaviviruses.^12^ Galidesivir is an iminoribitol *C*-nucleoside, which is converted to the triphosphorylated form by host kinases, and then incorporated into viral RNA by viral RNA-dependent RNA polymerases (RdRps). Primer extension assays with purified Hepatitis C virus (HCV) RdRp suggest that Galidesivir acts as a delayed chain terminator, although the mechanism remains undefined. In the case of HCV, it is reported that two further nucleotides are added subsequent to Galidesivir nucleotide incorporation, and then RNA synthesis ceases.^12^

**Figure 1:**
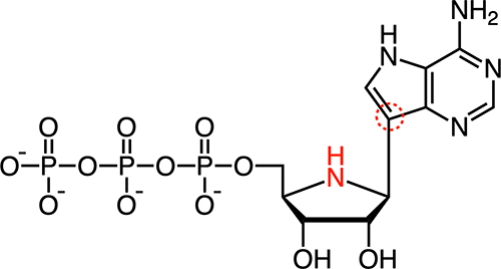
Galidesivir triphosphate (Gal-TP) is produced by the phosphorylation of Galidesivir by kinases in vivo. Red highlighting indicates sites of modification relative to adenosine triphosphate.

In cell-based assays, Galidesivir inhibits the replication of the flaviviruses YFV, JEV, DENV2, WNV, and TBEV with half maximal effective concentrations (EC_50_) in the low micromolar range.^12−14^ Galidesivir is also effective against YFV, TBEV, and ZIKV in rodent models of infection.^13,15,16^ In a nonhuman primate model, Galidesivir provided postexposure protection to ZIKV infection.^17^ This study also found that Galidesivir was not associated with reproductive toxicity at dosages of ≤75 mg/kg/day in pregnant rats and ≤25 mg/kg/day in pregnant rabbits, and that the antiviral crossed the placenta.^17^ In two phase I clinical trials of Galidesivir, no serious adverse effects were associated with the administration of the antiviral, and it was reported to be well-tolerated.^18^ Galidesivir is therefore a promising lead compound for the further development of therapeutics targeting flaviviruses, by inhibiting their RdRp activity.

The flaviviral RdRp is part of the multifunctional NS5 protein, fused to the C-terminus of a methyltransferase domain that is involved in RNA capping.^19^ Although the RdRp is active in the absence of the methyltransferase domain, full-length NS5 is required for efficient initiation and elongation.^20−22^ In vivo, flaviviral RdRps initiate RNA synthesis de novo, which involves the relatively slow formation of a dinucleotide primer from two incoming mononucleoside triphosphates.^22−26^

In all prior work, the effects of Galidesivir on flaviviral replication (Family *Flaviviridae*, Genus *Flavivirus*) have been assessed using cell-based antiviral assays. Results obtained with these assays reflect the cell-line-dependent efficiency with which host kinases convert Galidesivir into the active triphosphate form, as well as inhibition of the viral RdRp,^12−14^ and there may be off-target effects. The means by which Galidesivir might achieve delayed chain termination remain largely unexplored.

In this study, we chemically synthesized Galidesivir triphosphate (Gal-TP), enabling the direct measurement of its inhibitory effects against flaviviral RdRps, and the investigation of its mechanism of action. The inhibitory effects of Gal-TP on the RNA elongation activity of DENV2 and ZIKV NS5 were measured by the adaption of a continuous, fluorescent dye-based assay for double-stranded RNA formation.^27^ We introduced RNA primers into the assay system, allowing us to readily quantitate the effects of Gal-TP against the elongation activity of the polymerase. Subsequently, primer extension assays were used to gain a detailed understanding of the effects of Gal-TP on chain extension. Product formation was analyzed using denaturing gel electrophoresis with single-base resolution. Gal-TP incorporation does not cause delayed termination of RNA synthesis by the DENV2 NS5 polymerase, in contrast to what has been previously reported for the HCV RdRP.^12^

## Results

### Synthesis of Galidesivir Triphosphate

**Figure 2:**
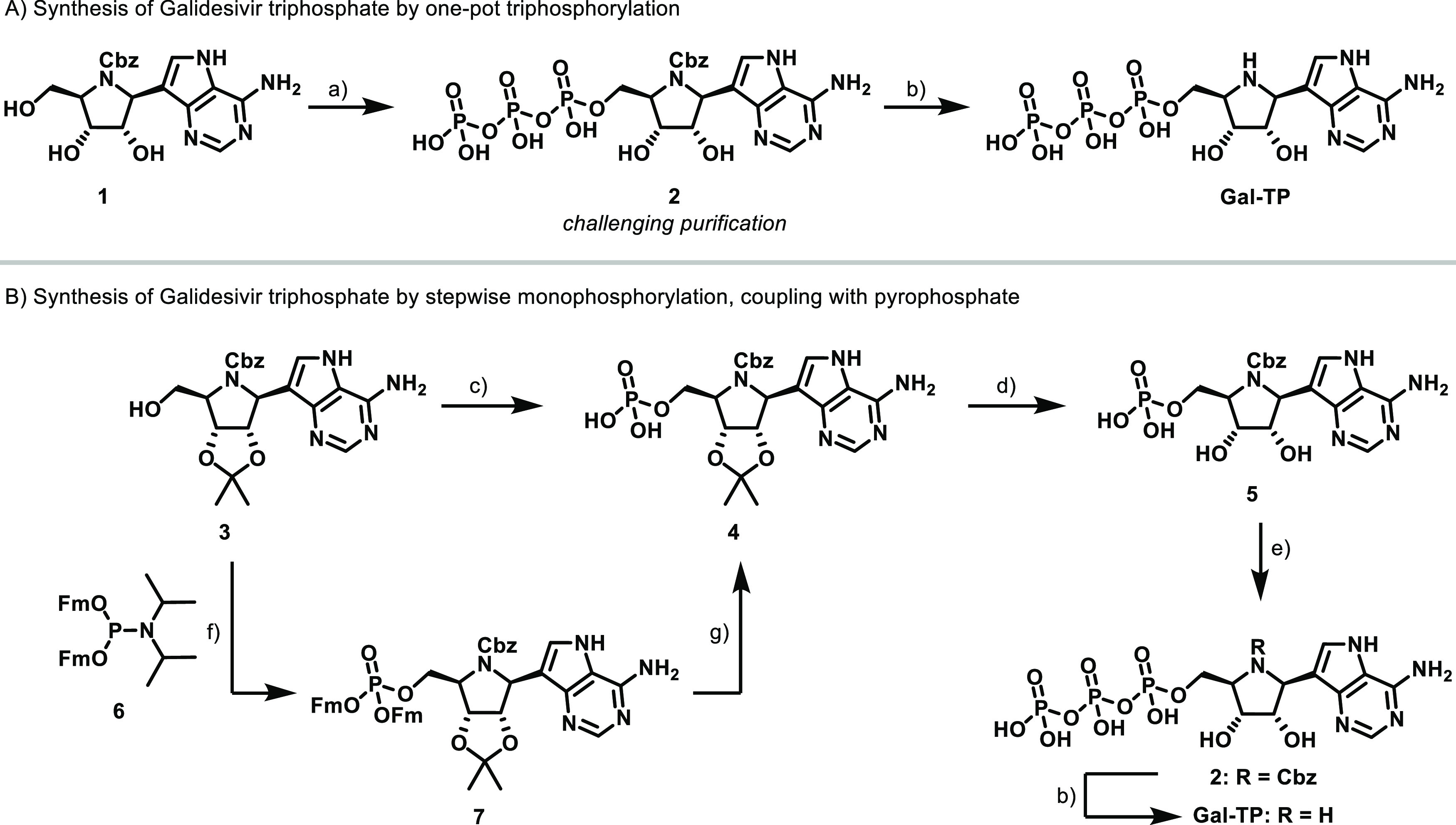
Synthesis of Galidesivir triphosphate. (A) Synthesis by one-pot triphosphorylation. (B) Synthesis by stepwise monophosphorylation, coupling with pyrophosphate. Reagents and conditions: (a) POCl_3_, (MeO)_3_PO, 0 °C, 3 h; then 2(*n*-Bu_3_N)·H_4_P_2_O_7_, *n*-Bu_3_N, MeCN, 2–3 h, 8–33% yield; (b) H_2_, Pd/C, H_2_O, rt, 1 h, 66% yield; (c) POCl_3_, (MeO)_3_PO, 0 °C for 1 h, rt for 2 h, then TEAB buffer (2 M aq.), 36% yield; d) TFA-DCM (1:1 v/v), rt, 20 h, 98% yield; (e) (i) CDI, DMF, rt, 3 h; (ii) 5% Et_3_N in MeOH-H_2_O (1:1 v/v), rt, 4 h; (iii) 2(*n*-Bu_3_N)·H_4_P_2_O_7_, *n*-Bu_3_N, DMF, rt, 20 h, then TEAB buffer (2 M aq.), 56% yield; (f) (i) phosphoramidite **6**, 1*H*-tetrazole, MeCN, rt, 25 min; (ii) *t*-BuOOH (70% w/w in H_2_O), 0 °C to rt, 50 min, 87% yield; (g) DMF-piperidine (10:1 v/v), rt, 3 h, 87% yield.

Initial attempts to prepare Galidesivir triphosphate commenced from the benzyl carbamate (Cbz)-protected iminoribitol **1** (Figure 2A). Cbz-protected substrate **1** was used under the rationale that the basic and nucleophilic iminoribitol nitrogen might interfere with triphosphorylation, and indeed, a recent report by Zhao et al. noted Galidesivir is not amenable to direct chemical triphosphorylation.^28^ Our chosen synthetic approach was highly analogous to that reported by Zhao et al., wherein synthesis of Galidesivir triphosphate was achieved in 15% overall yield from Galidesivir via a trifluoroacetamide-protected intermediate.^28^ Direct triphosphorylation of *N*-Cbz-protected Galidesivir (**1**) under Yoshikawa conditions delivered the expected triphosphate **2**;^29^ however, this method was low yielding and the reaction mixture contained impurities that were challenging to purge by chromatography.

In our hands, phosphorylation of a 2′,3′-di-*O*-isopropylidene-protected substrate **3**(30) proved more straightforward but necessitated the construction of the triphosphate via monophosphate **4** (Figure 2B). Installation of the monophosphate using phosphoryl chloride resulted in partial cleavage of the isopropylidene-protecting group. This was inconsequential, as the isopropylidene group was removed in the next step. We did, however, find that installation of the monophosphate using bisfluorenylmethyl phosphoramidite reagent **6** enabled higher-yielding synthesis of each intermediate in the sequence. Activation of monophosphate **5** with carbonyl diimidazole afforded a phosphorimidazolide intermediate, which on treatment with pyrophosphate gave triphosphate **2**. After hydrogenolysis of the Cbz-protecting group, Galidesivir triphosphate could be purified by ion-pair reversed-phase flash chromatography to deliver materials suitable for use in enzymatic assays. Although this synthetic approach is less concise than the recently reported literature method,^28^ it is higher yielding (27% yield from compound **3**, vs 18% yield from Galidesivir *N*-trifluoroacetamide), and this is particularly valuable in the context of the Galidesivir nucleoside, which itself is nontrivial to prepare.

### Production of Full-Length Flaviviral NS5 Proteins

NS5 proteins from Dengue-2 (DENV2) and Zika virus (ZIKV) were selected for testing of the inhibitory effects of Gal-TP, due to the global impacts of these viruses. Within the RdRp domain of NS5, there is near complete conservation of residues involved in substrate binding and catalysis.^31^ The sequences of DENV2 and ZIKV NS5 have 66% identity and 85% similarity when aligned (Figure S1).^32^

The DENV2 and ZIKV NS5 proteins were produced heterologously in *Escherichia coli* with an N-terminal poly-histidine tag appended. During the standardized purification protocol (see Methods) the poly-histidine tag was removed, as it is reported to have a deleterious effect on DENV2 RdRp activity.^33^ While DENV2 and ZIKV NS5 were purified free of significant contaminants, as assessed by SDS-PAGE (Figure S2), some proteolytic degradation was apparent, particularly for ZIKV NS5. As confirmed by mass spectrometry (data not shown), the major site of cleavage was the flexible linker between the methyltransferase and RdRp domains.

Analysis of purified DENV2 and ZIKV NS5 by size exclusion chromatography coupled to multiangle laser light scattering (SEC-MALLS) indicated that both proteins are predominantly monomeric in solution at concentrations less than 1 mg/mL (data not shown).

### Optimization of a Continuous Assay for Flaviviral NS5 RdRp Activity

We developed a medium-throughput assay to evaluate the inhibition of the primed RNA synthesis activity of flaviviral NS5s by Gal-TP and related nucleoside analogs. For this purpose, a continuous fluorescence assay reported by Sáez-Álvarez et al.,^27^ was adopted. The underlying principle of the assay is an enhancement in fluorescence of SYTO 9 upon binding to double-stranded RNA (dsRNA) in preference to single-stranded RNA (ssRNA). SYTO 9 is a cyanine dye;^34^ however, the exact structure is proprietary and its nucleic acid binding mechanism is not fully characterized. Notwithstanding, unlike many other dsRNA-binding dyes, SYTO 9 does not appear to significantly inhibit RdRp activity and hence is suitable for use in a continuous assay.^27^

The SYTO 9-based assay previously described measures de novo polymerase activity.^27^ However, de novo RNA synthesis by flaviviral RdRps involves a slow, rate-limiting initiation phase in which a dinucleotide is formed.^22−26^ For efficient initiation in vitro, high NTP concentrations must be used.^12^ In the presence of an RNA primer, the initiating step is artificially bypassed and the RdRp more rapidly transitions to processive RNA synthesis. A practical consequence is that the concentrations of nucleoside analogs required to generate dose–response curves in a primed assay are significantly reduced because the concentrations of competing cognate NTPs can be lower. For this reason, we elected to use a primed assay for a comparative analysis of the effects of Gal-TP on the elongation activity of the flaviviral polymerase. Although flaviviruses always initiate RNA synthesis de novo in a cellular setting,^22−26^ both de novo and primed reactions, performed in vitro with isolated polymerases and nongenomic templates, are highly simplified models for the replicative process that takes place in infected cells. Hence, care must be taken not to overinterpret the results of these in vitro assays in terms of the antiviral effects of Galidesivir in the host.

**Figure 3:**
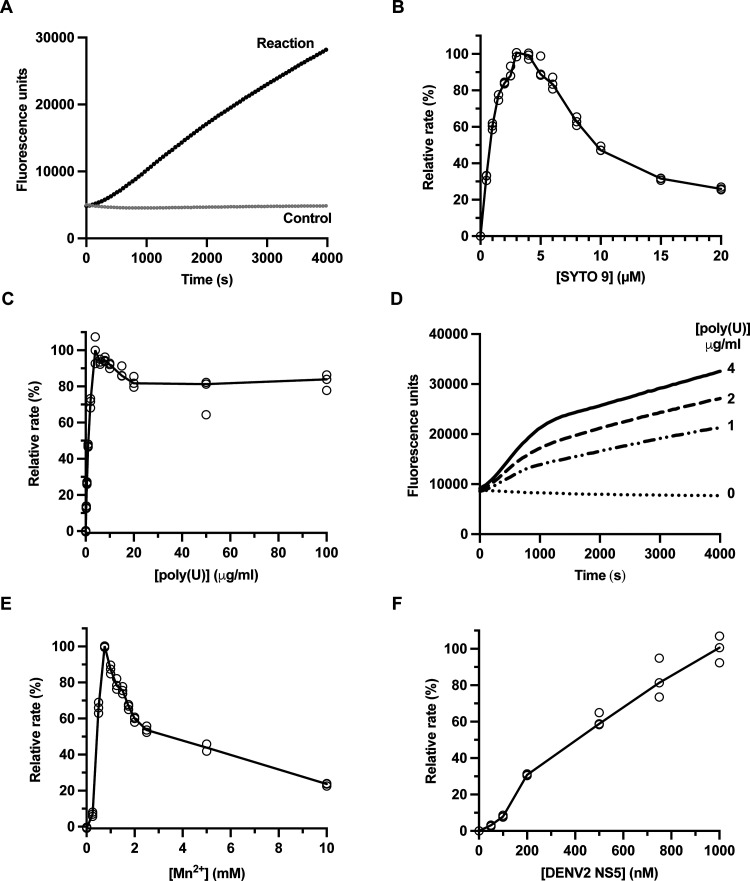
Optimization of the SYTO 9 fluorescence-based RdRp assay using DENV2 NS5. Unless otherwise stated reactions contained 200 nM DENV2 NS5, 2.5 mM MnCl_2_, 40 μg/mL poly(U), 2.5 μM SYTO 9 and were initiated by the addition of 0.5 mM ATP. The fluorescence excitation wavelength was 485 nm and the emission wavelength was 528 nm. (A) Reaction progress curve of a DENV2 NS5 catalyzed reaction, and a matched negative control lacking ATP. (B) Dependence of initial rate on SYTO 9 concentration (C) Dependence of initial rate on poly(U) template concentration (D) Biphasic reaction progress curves characteristically observed with poly(U) concentrations under 10 μg/mL (E) Dependence of initial rate on Mn^2+^ concentration. (F) Dependence of initial rate on enzyme concentration. All initial rates (panels B, C, E, and F) are reported relative to the maximum initial rate for that experiment, with the hollow circles showing the results of individual technical replicates, and the solid lines drawn through the median of each set of technical replicates.

To develop an assay for primed RNA synthesis, we first optimized conditions for de novo RNA synthesis by DENV2 NS5, using a poly(U) template. Subsequently, the effects of adding RNA primers were evaluated (see the following section). All assays were carried out at pH 7.5, consistent with previously reported activity optima,^21,35−38^ and at a temperature of 25 °C. An example of a de novo reaction progress curve for DENV2 NS5 following the initiation of the reaction by the addition of ATP is shown in Figure 3A, together with a matched negative control used to estimate the background fluorescence signal. Initial rates of fluorescence change were determined by a linear fit of background-corrected progress curves, excluding an apparent lag phase evident in the first 500–1000 s of the reaction.

A series of experiments were carried out to examine the effect of SYTO 9 dye, poly(U) template, divalent metal ion (Mn^2+^ or Mg^2+^), and enzyme concentration on the measured de novo rate for DENV2 NS5 (Figure 3). The SYTO 9 concentration previously used for assays of ZIKV RdRp^27^ (0.25 μM) did not provide a sufficient fluorescence signal in our assays. When the SYTO 9 concentration was varied (0.5–20 μM), the measured rate of fluorescence change increased sharply to a maximum at 3 μM SYTO 9 (Figure 3B). The signal decreased gradually at higher SYTO 9 concentrations, most likely due to an inner-filter effect.

Considering the effects of template concentration, the reaction rate underwent an initial steep increase, reaching a maximum at 4–10 μg/mL poly(U), but then effectively plateaued, declining only slightly at higher poly(U) concentrations (Figure 3C). This recapitulates previously reported behavior for the ZIKV RdRp domain.^27^ A template concentration of 40 μg/mL was selected for the subsequent assays, despite the slight attendant rate suppression, as the progress curves at low poly(U) concentrations (<10 μg/mL) were clearly biphasic (Figure 3D). The use of elevated poly(U) concentrations afforded a longer pseudolinear phase for the measurement of initial reaction rates.

The divalent metal ions Mn^2+^ and Mg^2+^ both support flaviviral RdRp activity in vitro. Significant rate enhancement is achieved by substituting Mn^2+^ for Mg^2+^.^39,40^ However, Mn^2+^ is also reported to be associated with increased misincorporation rates and copy-back activity.^41−45^ For DENV2 NS5, the maximum initial rate was several orders of magnitude faster in the presence of Mn^2+^ (Figure 3E) than in the presence of Mg^2+^ (Figure S3). Hence, Mn^2+^ was used exclusively in the assay. The initial rate was also strongly dependent on the Mn^2+^ concentration, with a well-defined maximum at 0.75 mM Mn^2+^ in the presence of 0.5 mM ATP (Figure 3E). As the free divalent metal ion concentration is highly dependent on the concentration of nucleoside triphosphates (NTPs), due to complex formation,^46^ the concentration of Mn^2+^ in each assay was systemically varied according to the maximum NTP concentration used (see Methods).

For DENV2 NS5, the initial reaction rate is a convex function of enzyme concentration at low concentrations (0–1 μM) with the relationship becoming approximately linear at higher concentrations (Figure 3F). This contrasts with the behavior expected of a simple monomeric enzyme, where the initial reaction rate should be strictly proportional to enzyme concentration. The nonlinear response at low enzyme concentrations could reflect a correlation between enzyme activity and self-association, as previously proposed.^47−50^ While isolated DENV2 NS5 is predominantly monomeric at the concentrations used in the assay, NS5 oligomerization could be promoted by the presence of RNA template, primer, and/or nucleotide substrates.

### Development of a Primed Assay for DENV2 NS5 RdRp Activity

Some prior analysis informed our experiments. A study of primed RNA elongation by DENV2 NS5 showed that a tetranucleotide primer was more efficiently incorporated than either shorter or longer primers, in an assay with a subgenomic RNA as a template.^24^ Similarly, for JEV NS5, the presence of a dinucleotide U2 primer led to higher rates of activity than a U10 primer in assays with a poly(A) template.^20^ Finally, in a study of the distantly related hepatitis C virus NS5B, pGG and pGGG primers were incorporated at higher efficiency than longer primers in assays with complementary heteropolymeric templates.^51^

**Figure 4:**
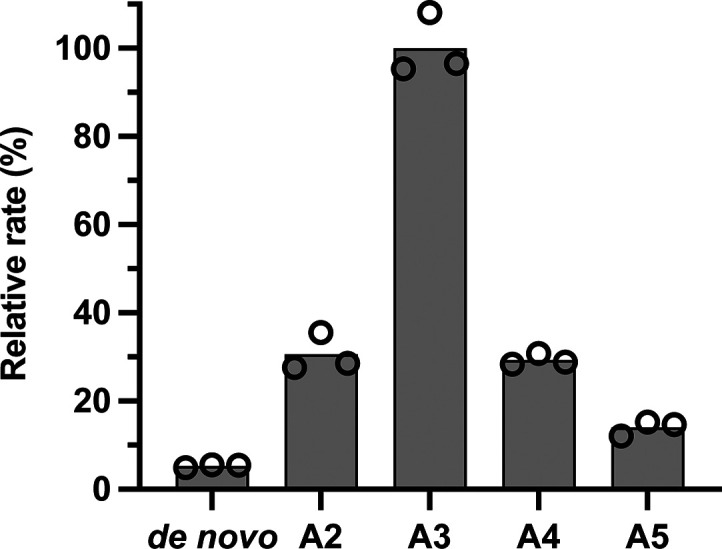
Enhancement of NS5 DENV2 activity by the addition of short poly(A) primers. Reactions contained 200 nM DENV2 NS5, 1.5 mM MnCl_2_, 40 μg/mL poly(U), 3 μM SYTO 9 and were initiated with 10 μM ATP. In addition, reactions contained either no primer (de novo reaction) or 10 μM of a non-5′-phosphorylated poly(A) primer. The data shown originate with primers A2 (ApA), A3 (ApApA), A4 (ApApApA), and A5 (ApApApApA). Gray bars represent the mean relative rate of three technical replicates, and hollow circles indicate the individual replicates.

Consequently, we tested the effects of poly(A) primers from two to ten nucleotides in length, on DENV2 NS5 RdRp activity with a poly(U) template (Figure 4). The A3 primer was most efficiently incorporated by DENV2 NS5, with a reaction rate 20-fold higher than in the de novo reaction at a primer concentration of 10 μM. The reaction rate in the presence of A2 and A4 primers was in each case 6-fold higher than the de novo reaction, while the reaction rate with the A5 primer was only 3-fold higher than the de novo reaction. With the use of longer primers (A6 to A10), rate enhancements decreased, and there were also substantial increases in background fluorescence due to annealing of the primers to the poly(U) template (data not shown).

Although the trinucleotide A3 primer is most efficiently incorporated by DENV2 NS5, this RNA primer must be custom synthesized and the cost of obtaining it in sufficient quantities for 96-well inhibition assays is currently high. Hence, the dinucleotide A2 primer was used for the inhibition assays described here, as it is readily sourced off-the-shelf at a lower cost.

### ATP-Dependence of DENV2 NS5 RdRp Activity in De novo and A2-Primed Reactions

**Figure 5:**
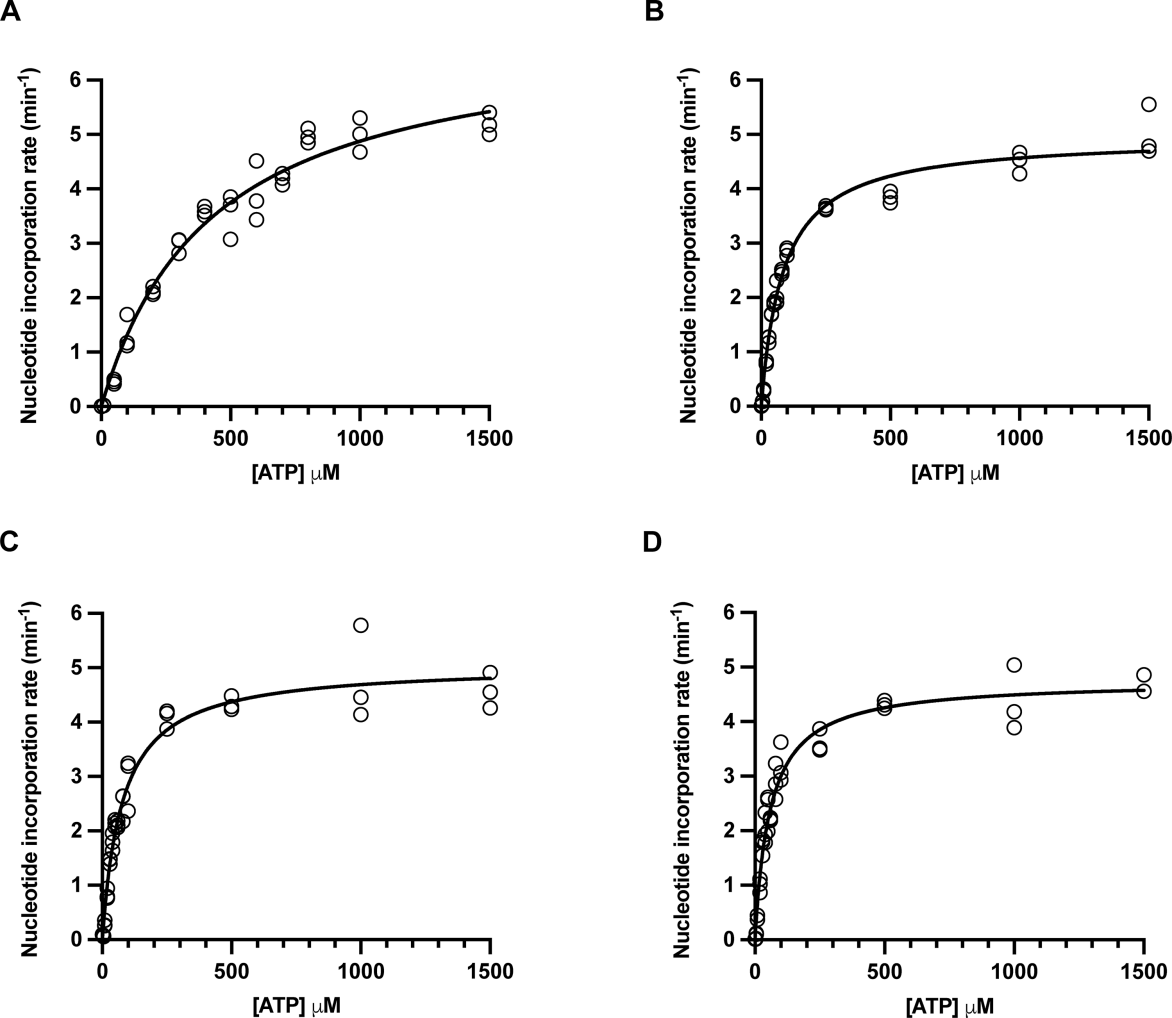
ATP dependence of DENV2 NS5 activity in the absence and presence of an A2 primer. The apparent *K*_M_ for ATP was determined in the absence of primer (de novo, panel A), and in the presence of A2 (ApA) primer at concentrations of 10, 20, and 40 μM (panels B, C, and D, respectively). Rates are reported as nucleotides incorporated per molecule of DENV2 NS5 per minute. Reactions contained 200 nM DENV2 NS5, 0 to 40 μM A2 primer, 40 μg/mL poly(U), 2.5 mM MnCl_2_, 3 μM SYTO 9, and were initiated with ATP at concentrations ranging from 0 to 1500 μM. Hollow circles show the experimentally estimated initial reaction rates, while solid lines show the fit of the data to the Michaelis–Menten equation.

The ATP-dependence of DENV2 NS5 RdRp activity was measured for both de novo and primed reactions with varying concentrations of the A2 RNA primer (Figure 5 and Table 1). For these experiments, rates of fluorescence change were converted to a nucleotide incorporation rate through the use of poly(A):poly(U) dsRNA standards (see Methods). This allowed absolute comparisons of nucleotide incorporation rates in the presence and absence of the primer.

**Table 1:** Effect of A2 Primer Concentration on the Apparent Kinetic Constants for DENV2 NS5

A2 primer concentration, μM	*K*_M_^app^ for ATP, (μM)	95% confidence interval of *K*_M_^app^ for ATP (μM)	*k*_cat_^app^ (min^–1^)	95% confidence interval of *k*_*c*at_^app^ (min^–1^)
0 (de novo)	430	350–530	7.0	6.4–7.6
10	85	76–96	5.0	4.7–5.1
20	78	67–90	5.1	4.8–5.3
40	59	50–68	4.8	4.5–5.0

For both de novo and primed reactions, the ATP-dependence data could be fit to the Michaelis–Menten equation to determine an apparent Michaelis constant *K*_M_ for ATP, and apparent rate constant *k*_cat_ for nucleotide incorporation (Figure 5 and Table 1). It should be noted that the reaction rates measured in the presence of primer result from both primed RNA synthesis and de novo initiation. At low ATP concentrations, the more efficient A2 primed reaction dominates, and at higher ATP concentrations, de novo initiation dominates.

In the absence of a primer (de novo reaction), *K*_M_^app^ for ATP was 430 μM (Figure 5A and Table 1). In the presence of 10 μM of A2 primer, *K*_M_^app^(ATP) was 85 μM, a 5-fold decrease relative to the de novo reaction (Figure 5B and Table 1). As the A2 primer concentration was further raised, *K*_M_^app^(ATP) incrementally decreased, down to a value of 59 μM with 40 μM of A2 primer (Figure 5C,D and Table 1). When the A2 primer was introduced, the apparent rate constant, *k*_cat_^app^, decreased 1.4-fold relative to the de novo reaction; however, there was no further change with increasing primer concentration (Table 1).

The Michaelis constant for de novo RNA synthesis by DENV2 NS5 is consistent with the *K*_M_^app^(ATP) of 560 ± 40 μM previously reported for de novo RNA synthesis by the ZIKV RdRp domain using a poly(U) template.^27^ A lower *K*_M_^app^ (ATP) of 32 ± 1 μM was reported for DENV2 NS5 in de novo initiation studies, using the first 20 nucleotides of the DENV2 antigenome as a template and with an Mn^2+^ cofactor.^22^ This is likely to reflect more efficient initiation on templates carrying the native CU-3′ sequence, relative to a homopolymeric poly(U) sequence. Submicromolar *K*_M_ values have been reported for all NTPs in processive RNA synthesis assays of DENV2 NS5 using long templates corresponding to the native 3′ UTR sequence of the DENV2 genome, and with an Mg^2+^ cofactor.^52^

For the Gal-TP inhibition assays, both the ATP and A2 primer concentrations were set to 20 μM. At these concentrations, the rate of DENV2 NS5-catalyzed nucleotide incorporation is approximately 10-fold faster than the de novo reaction with 20 μM ATP alone (Figure 5). This indicates that the reaction is dominated by primed RNA synthesis under these conditions. Although the reaction could be made even faster at higher A2 primer concentrations, the selected concentrations balance the ease of measurement with cost.

### Inhibition of DENV2 and ZIKV NS5 RdRp Activity by Gal-TP

The standardized assay conditions for testing inhibition of RdRp activity by Gal-TP are summarized in Table 2 (see also Methods). In all other experiments, enzymatic reactions were initiated by the addition of ATP, but for the inhibition assays the reaction was initiated by the addition of the NS5 protein. This avoids extended incubation of NS5 proteins with Gal-TP, which could conceivably result in the formation of ppp(Gal)p(Gal) dinucleotides. DENV2 NS5 catalyzes the formation of dinucleotide pppApG primers when incubated with ATP and GTP in the presence of Mn^2+^, even in the absence of a template.^26^

**Table 2:** Assay Components for NS5 RdRp Inhibition Assays

assay component	final concentration
MOPS/KOH, pH 7.5	50 mM
SYTO 9 dye	3 μM
MnCl_2_	2 mM
poly(U) template	40 μg/mL
A2 (ApA) primer	20 μM
ATP	20 μM
Gal-TP/3′-dATP	0–2000 μM/0 to 200 μM
NS5 protein (added to initiate reaction)	200 nM

**Figure 6:**
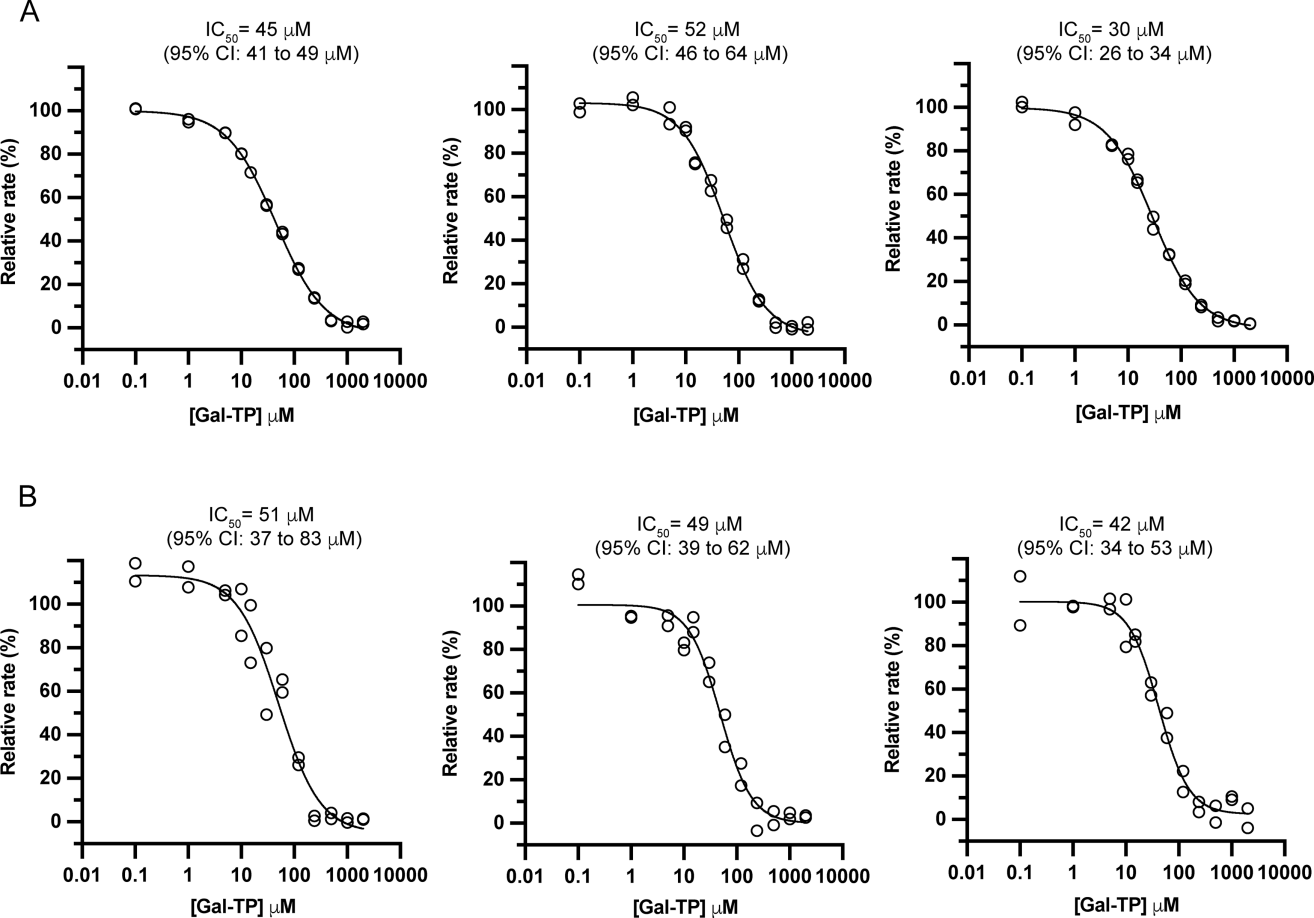
Inhibition of DENV2 (panel A) and ZIKV (panel B) NS5 RdRp activity by Gal-TP in A2 primed reactions. Dose–response curves for Gal-TP against NS5 RdRp activity were determined in three independent experiments as shown. Inhibition assay components are given in Table 2. Rates are given as a percentage relative to the uninhibited rate in the absence of Gal-TP. Data (hollow circles) were fit to a four-parameter logistic model (solid lines) to determine IC_50_ values.

Three independent dose–response experiments were carried out for DENV2 and ZIKV NS5 ([Gal-TP] = 0–2 mM) and the data fitted to a four-parameter logistic model (Figure 6). Gal-TP was equipotent against the RdRp activity of DENV2 and ZIKV NS5, with mean IC_50_ values of 42 ± 12 μM and 47 ± 5 μM, respectively (Table 3).

**Table 3:** Inhibition Constants for Gal-TP and 3′-dATP against DENV2 and ZIKV NS5 RdRp Activity (Poly(U) Template, 20 μM ATP, Mn^2+^ Cofactor)

nucleotide analog	NS5	IC50^a^ (μM, mean ± std. dev.)	slope parameter^a^ (*b*, mean ± std. dev.)
Gal-TP	DENV2	42 ± 12	–1.0 ± 0.1
Gal-TP	ZIKV	47 ± 5	–1.4 ± 0.2
3′-dATP	DENV2	0.48 ± 0.05	–1.2 ± 0.1
3′-dATP	ZIKV	0.06 ± 0.02	–0.9 ± 0.1

a Mean and standard deviation from three independent experiments. The four-parameter logistic model used for data fitting is detailed in the methods.

**Figure 7:**
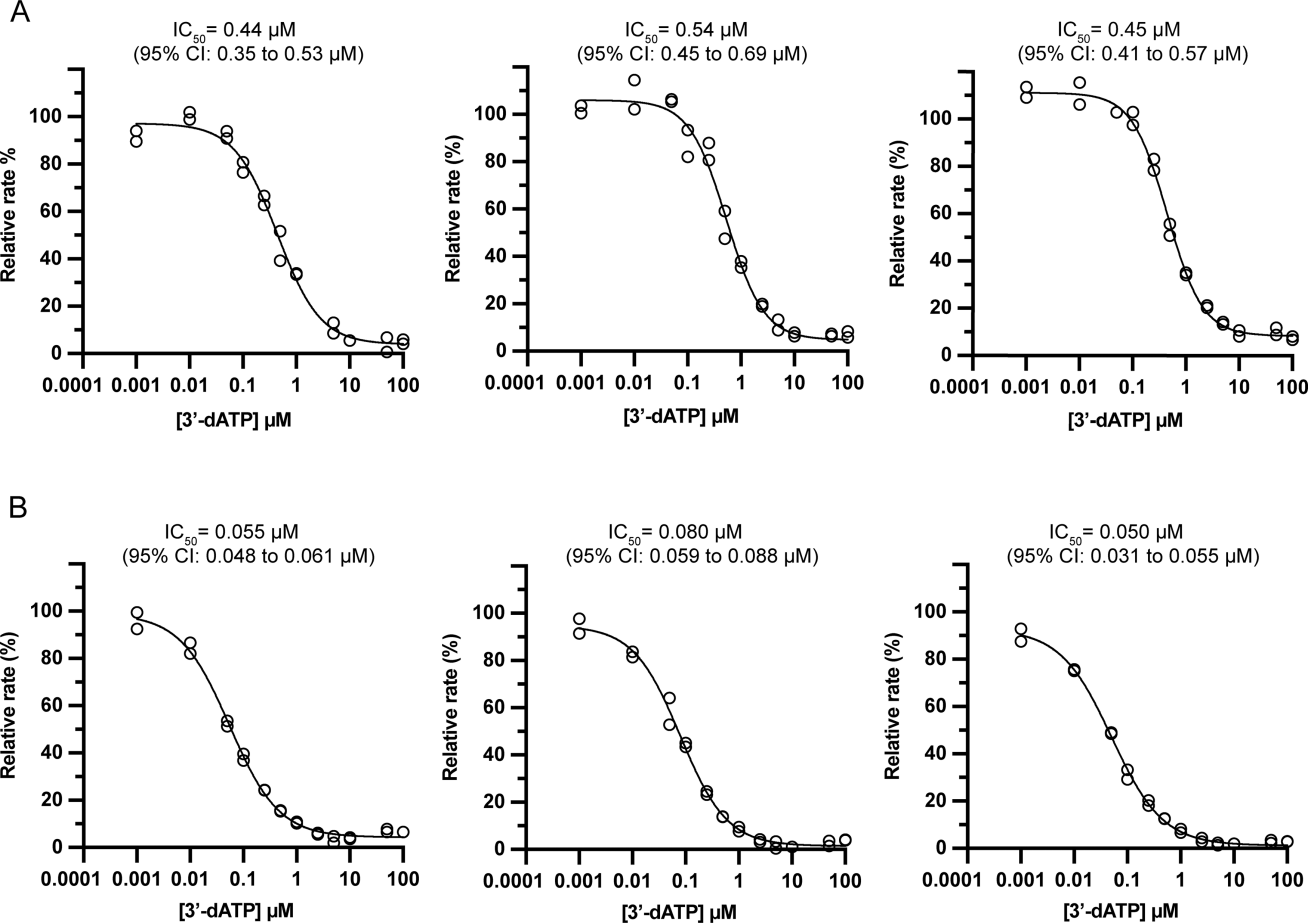
Inhibition of DENV2 (panel A) and ZIKV (panel B) NS5 RdRp activity by 3′-dATP in A2 primed reactions. Dose–response curves for 3′-dATP against NS5 RdRp activity were determined in three independent experiments as shown. Inhibition assay components are given in Table 2. Rates are given as a percentage relative to the uninhibited rate in the absence of 3′-dATP. Data (hollow circles) were fit to a four-parameter logistic model (solid lines) to determine IC_50_ values.

To validate the inhibition assay, the potency of the obligate chain terminator 3′-deoxyadenosine triphosphate (3′-dATP or cordycepin triphosphate) was also determined against DENV2 and ZIKV NS5 (Figure 7). Relative to Gal-TP, 3′-dATP was two orders of magnitude more potent against DENV2 NS5 and almost three orders of magnitude more potent against ZIKV NS5, with IC50 values of 0.48 ± 0.05 μM and 0.06 ± 0.02 μM, respectively (Table 3). Although direct comparisons are difficult due to the dependence of IC_50_ on assay conditions, these results are generally consistent with the low to submicromolar IC_50_ values reported previously for 3′-dATP against the processive RdRp activity of DENV2 and ZIKV NS5 in various discontinuous assays.^52−54^

### Gal-TP Promotes Stalling of DENV2 NS5 Immediately Prior to Incorporation, with a Very Strong Barrier to Incorporation at Consecutive Sites

The mechanism of action of Galidesivir has not been fully elucidated. For HCV NS5B RdRp, the proposed model is that subsequent to Galidesivir incorporation, up to two further nucleotides are added before the apparent termination of RNA synthesis (i.e., Galidesivir acts as a delayed chain terminator).^12^ However, this model has not been rigorously tested, and its applicability to other viral polymerases is unclear.

**Figure 8:**
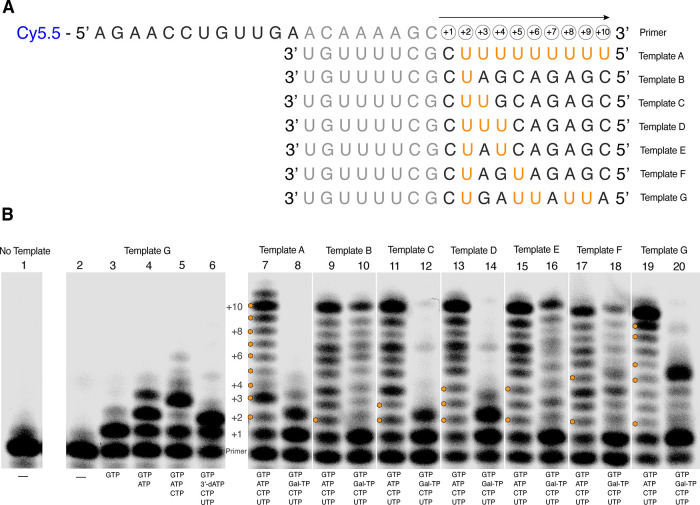
Effects of Galidesivir incorporation on DENV2 RdRp RNA synthesis investigated using a primer extension assay. (A) Sequences of the primer and templates used in the primer extension assay. Potential Galidesivir incorporation sites in the template sequences, assuming cognate Gal-U base-pairing, are highlighted in orange. (B) Results of the assay analyzed using denaturing polyacrylamide gel electrophoresis. Lane 1 is the primer by itself. Basic control reactions (lanes 2–6) were performed using template G in the presence of NTPs as indicated. Paired reactions to investigate the effects of Galidesivir incorporation (lanes 7–20) were performed using templates A–G. For each template (A–G), a control reaction with the natural NTPs (ATP, GTP, CTP, UTP) was compared to a reaction with ATP replaced by Gal-TP (Gal-TP, GTP, CTP, UTP). Adenosine incorporation sites in the reactions with the natural NTPs are highlighted by orange hexagons. The gel image has been split to separate the basic control reactions and paired experimental reactions for ease of interpretation. The unedited image is shown in Figure S6.

Denaturing gel electrophoresis was used to examine the RNA products formed by DENV2 and ZIKV NS5 in the presence or absence of Gal-TP, following the reaction for a fixed time period. As illustrated in Figure 8A, these experiments were conducted using one fluorescently labeled 20-nt RNA primer, and seven different unlabeled 18-nt RNA templates, with an 8-nt complementary region between primer and template. In preliminary experiments, significant misincorporation of natural NTPs was observed in control reactions containing Mn^2+^ (2 mM) as the only metal ion, consistent with previous reports.^41−45^ A mixture of Mn^2+^ (1.5 mM) and Mg^2+^ (0.5 mM) was found to minimize misincorporation, while still maintaining RNA synthesis activity at the levels required for the experiment (Figure S4). It was also observed that in the presence of ZIKV NS5, but not DENV2 NS5, substantial degradation of the fluorescent primer occurred, with a ladder of shorter RNA products evident (data not shown). This finding is consistent with the previously reported endonuclease activity of ZIKV NS5.^55^ Hence, DENV2 NS5 was used for all subsequent primer-extension experiments.

Prior to performing endpoint experiments, we executed an initial time course analysis with a single template, in the presence of the four natural NTPs (Figure S5). In addition to analyzing the reaction products, we also analyzed the fluorescently labeled primer, loaded at known concentrations. This analysis established two things central to the interpretation of the primer extension experiments. First, band intensity on polyacrylamide gels is a strictly increasing function of fluorescently labeled RNA concentration, over the ranges used in our experiments. Second, at the times selected for our endpoint experiments (30–90 min), the conditions are quasi-steady-state; hence, concentrations of all intermediates are approximately constant with time. This being the case, a significant build-up of an intermediate in the endpoint experiment reflects difficulty in progressing through that position in the template (i.e., the polymerase is pausing or stalling at that position).

Basic control reactions showed that the optimized endpoint assay was working as expected. Control reactions performed with NTP starvation are shown in Figure 8B, Lanes 2 through 5. In these cases, RNA synthesis was terminated at the expected positions (+1, Lane 3; +2 Lane 4; +3 Lane 5), with longer products produced in very limited amounts, showing that the effects of base misincorporation are not significant. A further control (Figure 8B, Lane 6) shows a reaction in which ATP is substituted with the obligate chain terminator 3′-dATP. This also caused immediate termination at the expected position (+2).

The template sequences were designed to determine the effect of Galidesivir nucleotide incorporation on RNA extension activity and probe the significance of sequence context. As detailed in Figure 8A, templates encoded potential Galidesivir nucleotide incorporation at single isolated sites, multiple isolated sites, or consecutive sites. For each template, annealed fluorescent primer/template substrates were incubated with DENV2 NS5, and with either the four natural NTPs (ATP, UTP, CTP, and GTP), constituting the positive control, or with Gal-TP added in place of ATP (Gal-TP, UTP, CTP, and GTP), constituting the treatment. Comparison of the treatment with matched positive control allowed the effects of Galidesivir nucleotide incorporation to be assessed.

Template A encoded incorporation of a single G nucleotide at the +1 site followed by nine consecutive A (or Galidesivir) nucleotides. This template is of most relevance for the inhibition assays with a poly(U) template described above. In the presence of the four natural NTPs and DENV2 NS5, production of a full-length 30-nt fluorescently labeled RNA occurred (Figure 8B, Lane 7). The production of slightly longer RNA products in small amounts is consistent with polymerase slippage occurring on the homopolymeric template sequence, as expected.^56^

On substitution of Gal-TP for ATP, production of the full-length product was effectively abolished, and instead, short RNA products truncated at the +1 and +2 sites accumulated (Figure 8B, Lane 8) The +1 product corresponds to the site immediately upstream of the first Galidesivir nucleotide incorporation site, suggesting stalling of the polymerase while attempting to incorporate Galidesivir. However, the substantial accumulation of the +2 product indicates that Galidesivir nucleotides can be incorporated into the nascent RNA chain. Some longer RNA products (+3, +4) are observed in very small amounts; however, because of the propensity for slippage on the homopolymeric template, their significance is unclear. Note that the +2 product carrying a Galidesivir nucleotide migrates slightly slower than the +2 product carrying an A nucleotide at the same position (Figure 8B, compare Lanes 7 and 8). This relative slowing of product migration upon Galidesivir nucleotide incorporation is a general phenomenon, which is observed throughout the experiment.

Further insights were gained from experiments with templates (B–F) encoding Galidesivir nucleotide incorporation at isolated or consecutive sites in a common background sequence. Template B encoded a single Galidesivir incorporation site at the +2 position, and this resulted in enhanced accumulation of a + 1 product in the presence of Gal-TP (Figure 8B, Lanes 9 and 10). There was no evidence of accumulation of a + 2 product and a full-length 30-nt RNA was also produced, albeit in reduced amounts relative to the reaction containing ATP. This suggests that once an isolated Galidesivir nucleotide is incorporated by DENV2 NS5, RNA extension can proceed relatively unhindered.

With templates encoding two (template C) and three (template D) consecutive Galidesivir incorporation sites, the product distribution resembles that seen for template A (Figure 8B, Lanes 11–14). RNA products accumulate at the +1 site, immediately upstream of the first Galidesivir incorporation site, and at the +2 site, corresponding to the first Galidesivir nucleotide incorporated. Almost no longer RNA products were observed. In contrast, with templates encoding two Galidesivir incorporation sites separated by one (template E) or two (template F) noncognate nucleotides, there was significant production of the full-length 30-nt RNA (Figure 8B, Lanes 15–18). Collectively, these results indicate a strong barrier to Galidesivir nucleotide incorporation at consecutive sites, which does not exist when the sites are isolated (i.e., separated by one or more noncognate nucleotides).

We also employed a template encoding one isolated site and two pairs of consecutive sites (template G), inserted in a different background sequence to templates (B–F). This resembles the template used to test the effects of Remdesivir on flaviviral RNA synthesis.^55^ Consistent with our prior results, RNA products accumulated at the +1 position, immediately upstream of the first isolated Galidesivir incorporation site (Figure 8B, Lanes 19 and 20). Accumulation of a +5 product is also observed, corresponding to the first Galidesivir nucleotide incorporated at the consecutive +5/+6 sites. Only very limited production of longer species is observed, consistent with a strong barrier to consecutive Galidesivir nucleotide incorporation.

**Figure 9:**
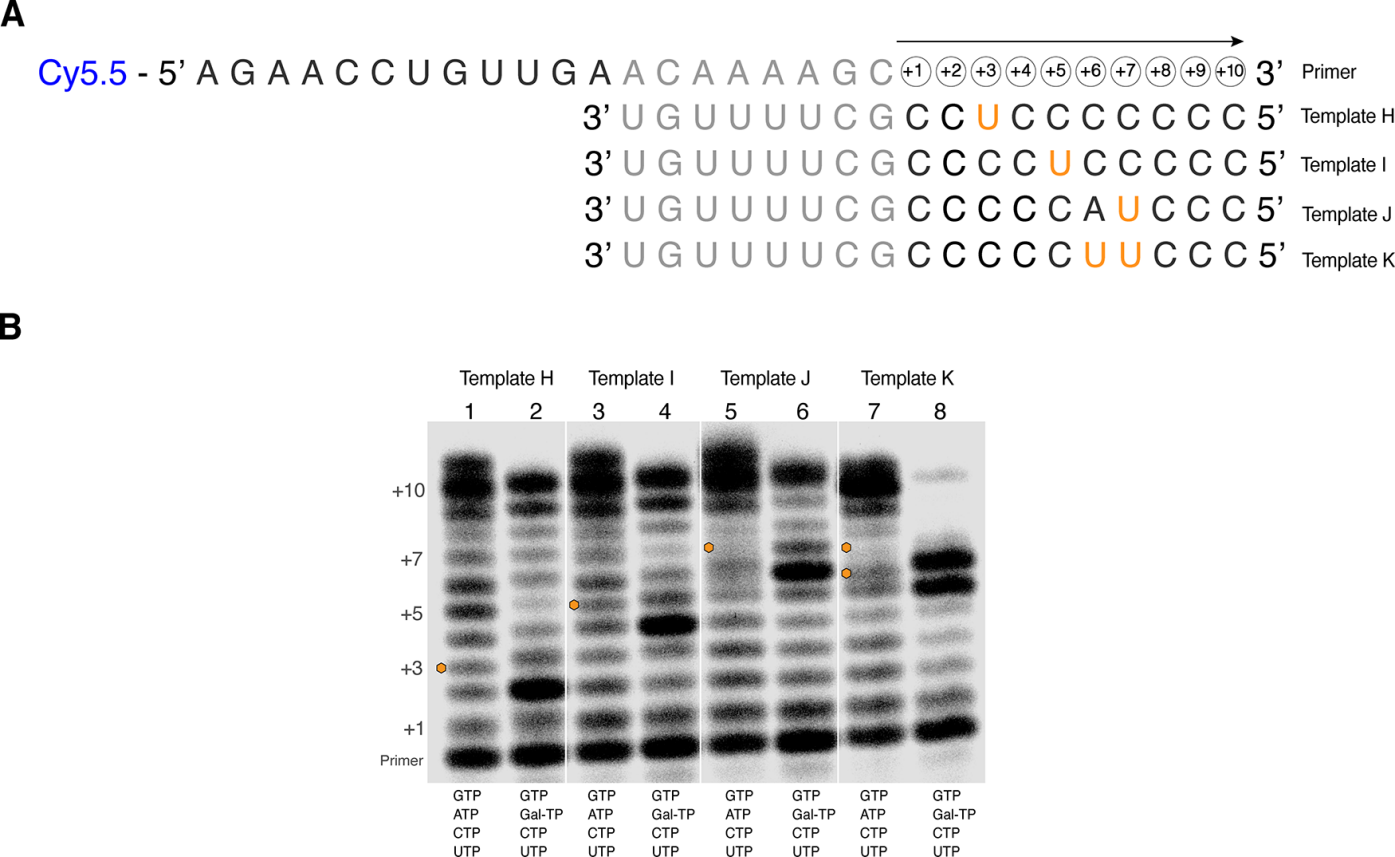
Effects of Galidesivir incorporation on DENV2 RdRp RNA synthesis investigated using a primer extension assay, with a simplified background sequence. (A) Sequences of the primer and templates used in the primer extension assay. Potential Galidesivir incorporation sites in the template sequences, assuming cognate Gal-U base-pairing, are highlighted in orange. (B) Results of the assay analyzed using denaturing polyacrylamide gel electrophoresis. Paired reactions to investigate the effects of Galidesivir incorporation (lanes 1–8) were performed using templates H–K. For each template (H–K), a control reaction with the natural NTPs (ATP, GTP, CTP, UTP) was compared to a reaction with ATP replaced by Gal-TP (Gal-TP, GTP, CTP, UTP). Adenosine incorporation sites in the reactions with the natural NTPs are highlighted by orange hexagons. The gel image has been split to separate the paired experimental reactions for ease of interpretation. The unedited image is shown in Figure S7.

To confirm the generality of our results, we performed primer-extension reactions with four further templates (H–K). These templates (Figure 9A) encoded Galidesivir nucleotide incorporation at a single isolated site (templates H–J), or two consecutive sites (template K). However, unlike the templates used previously (Figure 8A), the uracil bases were positioned within a simplified homopolymeric background sequence, and the initial uracil was moved away from the +2 position in the sequence. With the templates encoding a single incorporation site, the substitution of ATP with Gal-TP results in a general attenuation of full-length product synthesis (Figure 9B), and the relative buildup of intermediates at the position immediately upstream of the Galidesivir incorporation site (+2, template H; +4 template I; +6, template J). With template K, encoding two consecutive incorporation sites, the substitution of ATP with Gal-TP results in buildup of intermediates terminating immediately prior to the first incorporation site (+5), and with a single Galidesivir incorporated (+6), and the abolition of longer product synthesis (Figure 9B). Hence, the overall inhibitory effects of Gal-TP appear strongly dependent on the relative positioning of the incorporation sites, just as seen previously, but largely independent of the absolute positioning of the incorporation sites within the template sequence.

## Discussion

Given the impacts of flaviviruses on human health, and the likelihood of increased viral migration in a warming and interconnected world, there is a need to develop effective antiviral compounds. Nucleoside analogs such as Galidesivir are a promising avenue to pursue in antiviral development. In this study, we synthesized Galidesivir triphosphate (Gal-TP) and tested its inhibition of the RdRp activity of DENV2 and ZIKV NS5 using a dinucleotide-primed, continuous, fluorescence assay. Gal-TP had modest and equivalent micromolar potency against DENV2 and ZIKV NS5 RdRp activity. As far as we are aware, this is the first report of direct quantitative testing of the effects of Gal-TP on virally directed RNA synthesis. More generally, the primed assay described here usefully augments the de novo assay described previously^12^ and allows the inhibitory effects of nucleoside triphosphate analogs of modest potency to be readily compared.

Comparison of the in vitro potency of Gal-TP with that of other ATP analogs inhibiting flaviviral NS5 RdRp activity is complicated by the range of assay methods and conditions used in previous studies. An ATP analog carrying a 2′-*C*-ethynyl substituent on the ribose (7-deaza-2′-*C*-ethynyladenosine) had *K*_i_^app^ of 0.74 ± 0.072 μM against DENV2 NS5 in a radiometric assay using a template based on the native 3′-UTR sequence.^52^ RNA extension assay suggests that incorporation of this nucleotide analog leads to immediate chain termination.^52^ Two ATP analogs carrying 2′-*C*-methyl substituents on the ribose (2′-*C*-methyladenosine and 7-deaza-2′-*C*-methyladenosine) had IC_50_ values of 5.6 ± 0.07 and 7.9 ± 1.5 μM, respectively, against ZIKV RdRp with a poly(U) template and 2.5 μM ATP.^57^ NMR studies with poliovirus RdRp suggest that once incorporated the 2′-*C*-methyl substituent prevents incorporation of the following nucleotide by blocking closure of the RdRp active site.^58^

Most similar to Galidesivir, Remdesivir is a *C*-nucleoside analog of adenosine; however, Remdesivir carries a 1′-*C*-cyano substituent on the ribose ring. In a stopped fluorescence-based assay with 2 μM of a modified ATP substrate, Remdesivir triphosphate was reported to have low micromolar IC_50_ values against a range of flaviviral NS5s.^55^ Studies of the mechanism of action of Remdesivir using coronavirus polymerases show that three nucleotides are added following the incorporation of this nucleotide analog, and the polymerase then stalls due to a steric clash between the 1′-*C*-cyano substituent and a residue sidechain.^59−62^ However, this stalling is overcome in the presence of high concentrations of NTPs.^59^

The template-directed primer extension assays reported in this study (Figures 8 and 9) suggest how Gal-TP interferes with RNA synthesis by DENV2 NS5. The persistent accumulation of RNA products truncated immediately upstream of an isolated Galidesivir incorporation site shows that DENV2 NS5 stalls while attempting to add a Galidesivir nucleotide to the nascent RNA chain (i.e., the polymerase has difficulty incorporating Galidesivir and, therefore, cannot progress rapidly through this position in the template). Stalling could be promoted through compromise of either nucleotide binding or catalysis, and our data do not address this question. However, once a Galidesivir nucleotide is successfully added, the polymerase can proceed to synthesize full-length RNA products. There is no evidence for delayed chain termination following Galidesivir incorporation.

The primer extension assays also demonstrate an additional feature of Galidesivir-mediated inhibition. DENV2 NS5 is strongly hindered from incorporating Galidesivir nucleotides at consecutive sites in the nascent RNA chain (i.e., whenever there are consecutive uracil bases in the template sequence: Figure 8, templates A, C, D, and G, Figure 9, template K). RNA products terminating at the first encoded Galidesivir nucleotide accumulate, and the synthesis of longer RNA products is dramatically reduced relative to matched controls where ATP is incorporated. This effect is clearly demonstrated by comparing the results obtained with template C (where there are two consecutive Galidesivir incorporation sites) and templates E and F (where there are two isolated Galidesivir incorporation sites). In other words, when the polymerase is presented with consecutive incorporation sites and ATP is absent, polymerase stalling is near complete after incorporation of the first Gal-TP. These data are consistent with Galidesivir causing a distortion in the dsRNA that hinders the incorporation of a subsequent Galidesivir nucleotide. Sequence-dependent modulation of inhibitory activity has been observed for other nucleoside analogs that directly interfere with RNA synthesis.^63^

Caution is needed when inferring the effect of Galidesivir on viral replication from the results of these simple in vitro experiments, which were performed with isolated polymerases and nongenomic templates. In vivo, Gal-TP must compete with the millimolar intracellular concentrations of ATP.^64^ As Gal-TP has only modest potency against the elongation activity of DENV2 and ZIKV NS5 in our in vitro assays (Figure 6), Gal-TP-promoted stalling is readily relieved in the presence of ATP. Hence, in a cellular setting, the strong barrier to the incorporation of Galidesivir at consecutive sites, observed in the absence of ATP in vitro, is unlikely to result in early chain termination. In addition, although Galidesivir incorporation does not lead to delayed chain termination in our in vitro assay, downstream effects of Galidesivir incorporation on viral RNA replication cannot be ruled out in vivo. For example, secondary inhibitory effects of Remdesivir-incorporated RNA templates on replication by the coronavirus RdRp have been reported.^65^

Galidesivir has previously been proposed to act as a delayed chain terminator, based on primer extension assays performed in vitro with purified Hepatitis C virus (HCV) RdRp.^12^ However, our primer extension assays show that the reported termination of RNA synthesis two nucleotides after the addition of Galidesivir does not occur during DENV2 NS5-catalyzed RNA elongation. In contrast, Gal-TP acts to inhibit DENV2 NS5 polymerase activity prior to incorporation. Most likely, it functions as a competitive inhibitor of chain elongation, with respect to ATP. Further studies are required to determine if this is the source of the inhibitory effect of Galidesivir against DENV2, and other members of genus *Flavivirus*, observed in cell-based assays and animal models of infection.^13−17^

Iminoribitol *C*-nucleosides, such as Galidesivir, are a promising class of antiviral compounds. Unlike previously characterized adenosine analogs that cause steric clashes with the viral polymerase, Galidesivir does not carry additional bulky substituents, and hence, its inhibitory activity is likely due to very subtle differences in geometry or base pairing, relative to adenosine. Both structural and further biochemical studies are needed to understand why Gal-TP promotes stalling of the DENV2 NS5 polymerase, and why consecutive incorporation of Galidesivir nucleotides is particularly unfavorable. A deeper understanding of the mechanism of action of Galidesivir will assist the rational design of new nucleoside analog-based antivirals for the future treatment of endemic and emerging flaviviruses.

## Methods

### Synthesis of Galidesivir Triphosphate

Full details of the synthesis and characterization of Galidesivir triphosphate are given in the Supporting Information.

### Protein Production and Purification

Genes encoding the full-length NS5 proteins from DENV2 (GenBank accession no. NC001474, UniProt accession no. P29990), and ZIKV (GenBank accession no. NC012532, UniProt accession no. Q32ZE1) were commercially synthesized and cloned into plasmid pUC57 by GenScript Biotech. NS5 coding sequences were subsequently transferred into expression plasmid pET-15b (Novagen) using standard molecular biology procedures (Figure S8). These expression constructs appended an N-terminal poly-histidine tag to the NS5 protein, with an interleaving tobacco etch virus (TEV) protease cleavage site to facilitate tag removal.

Production of NS5 proteins was carried out using an autoinduction protocol,^66^ in *E. coli* Rosetta 2 (DE3) cells (Novagen). Chemically competent Rosetta 2 (DE3) were transformed with the relevant NS5 expression plasmid. Precultures (10 mL) inoculated from a single colony of the transformed bacteria were grown overnight at 30 °C in a noninducing minimal medium, MDG, supplemented with appropriate antibiotics. Overexpression was carried out by inoculating 5 mL of the preculture into 500 mL of autoinducing medium, ZYM-5052 in baffled flasks. Cultures were maintained at 37 °C for 2.5 h and then 18 °C for 20 h, with shaking at 160 rpm throughout. Bacteria were pelleted by centrifugation and stored at −20 °C until further processed.

To purify NS5 proteins, cell pellets were thawed, and the bacteria resuspended in lysis buffer (10 mL per g of cell pellet) comprising 50 mM potassium phosphate, pH 7.5, 300 mM NaCl, 10% glycerol and 5 mM β-mercaptoethanol. HEWL Lysozyme (50 μg/mL, Sigma Aldrich), Bovine pancreatic DNase I (10 μg/mL, Sigma), and an EDTA-free protease inhibitor cocktail tablet (cOmplete, Roche Diagnostics) were added to the cell suspension before incubating on ice for 15 min. Cells were lysed using a continuous cell disruptor (M-110P Microfluidizer, Constant Systems Ltd). The lysate was centrifuged at 20,000×*g* for 30 min at 4 °C to pellet cellular debris, and the supernatant was loaded onto TALON IMAC resin (Takara Bio) pre-equilibrated with the lysis buffer, under gravity flow. NS5 proteins were eluted from the IMAC resin using lysis buffer supplemented with 300 mM imidazole. The eluted protein was dialyzed overnight at 4 °C against the lysis buffer, adding recombinant TEV protease to a final concentration of 0.1 mg/mL during the dialysis step.

The protein was subsequently passed over a fresh bed of TALON IMAC resin, pre-equilibrated with lysis buffer, to remove uncleaved NS5 protein that retained the poly-histidine tag. The column flow-through was spin concentrated and subjected to size exclusion chromatography (SEC) Superdex 200 media (Cytiva). The SEC column was pre-equilibrated with 20 mM MOPS/KOH pH 7.5, 300 mM NaCl, 10%(v/v) glycerol and 1 mM TCEP-HCl, and the protein was eluted isocratically in this buffer. Fractions containing the purified NS5 protein were pooled and concentrated to 0.2–1 mg/mL. The protein was aliquoted; flash-frozen in liquid nitrogen; and stored at −80 °C until subsequent use. Protein concentrations were calculated from UV absorbance measurements, using molar absorbance coefficients estimated from the protein sequence.^67^

### Size Exclusion Chromatography Coupled to Multiangle Laser Light Scattering

Size exclusion chromatography coupled to multiangle laser light scattering (SEC-MALLS) analysis of the purified NS5 proteins was performed using a Superdex 200 10/300 column (Cytiva) connected to an Ultimate 3000 HPLC system (Thermo Fisher Scientific) equipped with in-line MALLS (Polymer Standards Service SLD7000) and differential refractive index (Shodex RI-101) detectors. The SEC column was equilibrated with 20 mM MOPS/KOH pH 7.5, 300 mM NaCl, 10% (v/v) glycerol, 3 mM sodium azide and 1 mM TCEP-HCl. NS5 proteins were loaded at concentrations of 0.45 and 0.9 g/L, and eluted isocratically. The mass averaged molar mass of the eluted proteins was determined from the refractive index and light scattering measurements using the PSS WinGPC UniChrom 8.1 software, under the assumption of Rayleigh scattering. A constant refractive index increment of 0.186 was used to estimate all protein concentrations, with the MALLS detector calibrated using a bovine serum albumin solution.

### SYTO 9 Fluorescence-Based RdRp Assays

A fluorescence-based assay adapted from a study by Sáez-Álvarez and coworkers was used to measure RdRp activity.^27^ All reagents and buffers were prepared in nuclease-free water. The assays were carried out in 96-well black, flat-bottom microplates (Greiner). Assay mixtures (100 μL) were prepared as detailed below, and the enzymatically catalyzed reactions were typically initiated by the addition of ATP. Fluorescence was recorded for 1 h on a Biotek Synergy HTX plate reader (Agilent) with excitation and emission filters of wavelength 485/20 and 528/20 nm, respectively.

The background fluorescence signal for each reaction was estimated from a matched negative control, from which only ATP was omitted. The initial rates of fluorescence increase were measured by linear fitting of background-corrected progress curves, excluding the initial region of the curve if there was any apparent lag phase (duration 500–1000 s). Technical duplicates or triplicates were collected for each assay condition. Data analysis, including background subtraction, linear fitting to determine initial rates, and nonlinear model fitting to estimate apparent kinetic constants and IC_50_ values, was carried out using GraphPad Prism version 9.0.

### Optimization of De novo Reaction Conditions for DENV2 NS5

Unless otherwise stated, the assay mixtures for initial optimization of the de novo reaction contained 50 mM MOPS/KOH pH 7.5, 2.5 mM MnCl_2_, 2.5 μM SYTO 9 (Thermo Fisher), 40 μg/mL polyuridylic acid (poly(U), Sigma), 200 nM DENV2 NS5, and were initiated by the addition of 500 μM ATP (New England Biolabs).

### Testing the Effects of Primer Addition for DENV2 NS5

Unless otherwise stated, the assay mixtures for primed reactions contained 50 mM MOPS/KOH pH 7.5, 1.5 mM MnCl_2_, 3 μM SYTO 9, 40 μg/mL poly(U), 200 nM DENV2 NS5 and RNA primer. Reactions were initiated with ATP, as detailed below.

For establishing the most efficiently incorporated primer, non-5′-phosphorylated poly(A) primers varying in length from A2 to A10 were obtained commercially. A2 (ApA) RNA dinucleotide primer was purchased from Jena Bioscience. A3 to A10 primers were synthesized by GenScript Biotech. Comparative assays were run as described above with 10 μM RNA primer (A2 to A10) present, or no primer for the de novo reaction. Reactions were initiated by the addition of 10 μM ATP.

Experiments to measure the effect of A2 primer concentration on the apparent *K*_M_ and *V*_max_ were performed by measuring the initial rate at ATP concentrations ranging from 0 to 1500 μM and at A2 concentrations from 0 to 40 μM. ATP concentrations did not exceed 1.5 mM to prevent depletion of free Mn^2+^, which would result in large rate variations.

The ATP-dependence data were fitted to the Michaelis–Menten equation:
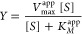
1where *Y* is the reaction rate, [*S*] is the substrate concentration, *V*_max_^app^ is the maximal reaction rate, and *K*_M_^app^ is the substrate concentration at half the maximal reaction rate.

The apparent turnover number, *k*_cat_^app^, is defined as:
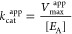
2where [*E*_A_] is the concentration of NS5 enzyme active sites.

For ATP-dependence experiments, the rate of fluorescence change was converted to the rate of nucleotide incorporation using a standard curve generated with the double-stranded RNA poly(A)-poly(U) (Sigma Aldrich), which is the reaction product detected by SYTO 9. The poly(A)-poly(U) was added at 0 to 8 μg/mL concentrations to assay mixtures containing the same components as the reaction mixtures, with the exception of ATP. The fluorescence of the standard solutions was read after a 2000 s incubation in the plate reader to allow for signal stabilization. Fluorescence was plotted against the effective concentration of A:U base pairs in each standard sample, and a linear fit was used to determine the fluorescence change per nucleotide incorporated (an example standard curve is presented in Figure S9). After converting raw reaction rates to nucleotides incorporated per minute, rates were normalized by the enzyme concentration, giving final reaction rates in terms of nucleotides incorporated per NS5 molecule per minute (min^–1^).

### Inhibition Assays with Nucleoside Triphosphate Analogs

Assay mixtures for measuring inhibitory effects of Gal-TP on DENV2 and ZIKV NS5 contained 50 mM MOPS/KOH, pH 7.5, 2.0 mM MnCl_2_, 3 μM SYTO 9, 40 μg/mL poly(U), 20 μM A2 primer, 20 μM ATP, 0 to 2 mM Gal-TP, and were initiated by addition of 200 nM DENV2 NS5. The concentration of Gal-TP did not exceed 2 mM to prevent the formation of an insoluble complex with Mn^2+^ (data not shown). Inhibition assays with 3′-deoxyATP were carried out in the same fashion but with 0 to 200 μM 3′-deoxyATP in place of Gal-TP.

Reaction rates were measured and expressed as a percentage of the uninhibited rate for each NS5. The data were fit to an empirical four-parameter logistic model:^68^
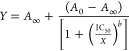
3where *Y* is the reaction rate, *X* is the inhibitor concentration, *A*_0_ and *A_∞_* are the asymptotic values for the reaction rate at very low and very high inhibitor concentrations, respectively, IC_50_ is the half maximal inhibitory concentration, and *b* is a slope parameter.

### Primer Extension Assays

Primer extension assays were performed using a fluorescently labeled primer,^55^ and an array of templates custom synthesized by Sigma Aldrich (Table 4). Reaction mixtures (20 μL) contained 50 mM MOPS-KOH pH 7.5, 200 nM primer, 400 nM template, 0.5 mM MnCl_2_, 1.5 mM MgCl_2_ and 400 nM DENV2 NS5. Reactions were initiated by adding NTPs or NTP analogs (each at 10 μM) as dictated by the experiment and allowed to progress for 30–90 min at 30 °C. Reactions were quenched by adding 20 μL of stop buffer (80% formamide, 50 mM Na.EDTA (pH 8.0), 10% glycerol, and a trace quantity of bromophenol blue). Samples were denatured at 95 °C for 10 min and immediately placed on ice. The RNA products were separated on a 20% polyacrylamide gel containing 7 M urea. Electrophoresis was performed in 1× TBE buffer at 55 °C using an omniPAGE WAVE Maxi system (Cleaver Scientific), under constant voltage conditions (1 h at 350 V followed by 2.5 h at 450 V). The gel was subsequently fixed in 1× TBE buffer supplemented with 5% ethanol and 5% methanol for 10 min and imaged using an LI-COR Odyssey CLx fluorescence imager.

**Table 4:** Sequences of the Primer and Templates Used for the Primer Extension Assay

primer	*Cy5.5*–5′ AGAACCUGUUGAACAAAAGC 3′
Template A	5′ UUUUUUUUUCGCUUUUGU 3′
Template B	5′ CGAGACGAUCGCUUUUGU 3′
Template C	5′ CGAGACGUUCGCUUUUGU 3′
Template D	5′ CGAGACUUUCGCUUUUGU 3′
Template E	5′ CGAGACUAUCGCUUUUGU 3′
Template F	5′ CGAGAUGAUCGCUUUUGU 3′
Template G	5′ AUUAUUAGUCGCUUUUGU 3′
Template H	5′ CCCCCCCUCCGCUUUUGU 3′
Template I	5′ CCCCCUCCCCGCUUUUGU 3′
Template J	5′ CCCUCCCCCCGCUUUUGU 3′
Template K	5′ CCCUUCCCCCGCUUUUGU 3′

## Supporting Information Available

Section A: Sequence alignment of the DENV2 and ZIKV NS5 proteins (Figure S1); SDS-PAGE analysis of purified NS5 proteins (Figure S2); comparison of DENV2 NS5 RdRp activity in the presence of Mn^2+^ and Mg^2+^ ions (Figure S3); optimization of divalent metal ion concentration for primer extension assays (Figure S4); time course of a primer extension reaction (Figure S5); unedited gel images for Figures 8 and 9 in the main text (Figure S6 and S7, respectively); details of NS5 gene insertion into the pET15b(+) expression vector (Figure S8); standard curve showing SYTO9 fluorescence versus poly(A):poly(U) concentration (Figure S9); and Section B: Experimental details for chemical synthesis of Galidesivir triphosphate (PDF)

## Abbreviations

DENV: dengue virus
EDTA: ethylenediaminetetraacetic acid
Gal-TP: galidesivir triphosphate
HEWL: hen egg-white lysozyme
IMAC: immobilized metal affinity chromatography
JEV: Japanese encephalitis virus
MALLS: multi-angle laser light scattering
MOPS: 3-(*N*-morpholino)propanesulfonic acid
RdRp: RNA-dependent RNA polymerase
SEC: size exclusion chromatography
TBEV: tick-borne encephalitis virus
TCEP: tris(2-carboxyethyl)phosphine
TEV: tobacco etch virus
WNV: West Nile virus
YFV: yellow fever virus
ZIKV: Zika virus
