# Supplementary material for: Galidesivir Triphosphate Promotes Stalling of Dengue-2 Virus Polymerase Immediately Prior to Incorporation
Source: ACS Infect Dis. 2023 Jul 24;9(8):1658–73. doi: 10.1021/acsinfecdis.3c00311 (PMC10739630; doi:10.1021/acsinfecdis.3c00311)
Supplement: Supplementary file 1 — id3c00311_si_001.pdf [file id3c00311_si_001.pdf]

Supplementary Information

Galidesivir triphosphate promotes stalling of dengue-2 virus polymerase immediately prior to incorporation.

Sandesh Deshpande[a], Wenjuan Huo[a], Rinu Shrestha[b,c], Kevin Sparrow[b], James M. Wood[b,c], Gary B. Evans[b,c], Lawrence D. Harris[b,c], Richard L. Kingston[a,c*], Esther M. M. Bulloch[a,c]

[a] School of Biological Sciences, University of Auckland, Auckland 1010, New Zealand

[b] Ferrier Research Institute, Victoria University of Wellington, 69 Gracefield Rd, Lower Hutt 5010, New Zealand.

[c] Maurice Wilkins Centre for Molecular Biodiscovery, University of Auckland, Auckland 1010, New Zealand

*Corresponding author: rl.kingston@auckland.ac.nz

Section A: Supplementary Figures

Supplementary Figures 1 to 9.

Section B: Chemical synthesis of Galidesivir triphosphate

Experimental details and compound characterization data.

Section A: Supplementary Figures

```
DENV2_NS5       GTGNIGETLGEKWKSRLNALGKSEFQIYKKSGIQEVDRTLAKEGIKRGE-TDHHAVSRGS  59
ZKV_NS5         -GGGTGETLGEKWKARLNQMSALEFYSYKKSGITEVCREEARRALKDGVATGGHAVSRGS  59
                  *. *********:*** :.  **  ****** ** *  *:..:* *  *. *******

DENV2_NS5       AKLRWFVERNMVTPEGKVVDLGCGRGGWSYYCGGLKNVREVKGLTKGGPGHEEPIPMSTY  119
ZKV_NS5         AKIRWLEERGYLQPYGKVVDLGCGRGGWSYYAATIRKVQEVRGYTKGGPGHEEPMLVQSY  119
                **:**: **. : * ****************.. :::*:**:* **********: :.:*

DENV2_NS5       GWNLVRLQSGVDVFFIPPEKCDTLLCDIGESSPNPTVEAGRTLRVLNLVENWLNNN-TQF  178
ZKV_NS5         GWNIVRLKSGVDVFHMAAEPCDTLLCDIGESSSSPEVEETRTLRVLSMVGDWLEKRPGAF  179
                ***:***:******.:  * ************ .* **  ******.:* :**::.   *

DENV2_NS5       CIKVLNPYMPSVIEKMEALQRKYGGALVRNPLSRNSTHEMYWVSNASGNIVSSVNMISRM  238
ZKV_NS5         CIKVLCPYTSTMMETMERLQRRHGGGLVRVPLCRNSTHEMYWVSGAKSNIIKSVSTTSQL  239
                ***** **  :::*.** ***::**.*** **.***********.*..**:.**.  *::

DENV2_NS5       LINRFTMRYKKATYEPDVDLGSGTRNIGIESEIPNLDIIGKRIEKIKQEHETSWHYDQDH  298
ZKV_NS5         LLGRMDGPRRPVKYEEDVNLGSGTRAVASCAEAPNMKIIGRRIERIRNEHAETWFLDENH  299
                *:.*:    : ..** **:****** :.  :* **:.***:***:*::**  :*. *::*

DENV2_NS5       PYKTWAYHGSYETKQTGSASSMVNGVVRLLTKPWDVVPMVTQMAMTDTTPFGQQRVFKEK  358
ZKV_NS5         PYRTWAYHGSYEAPTQGSASSLVNGVVRLLSKPWDVVTGVTGIAMTDTTPYGQQRVFKEK  359
                **:*********:   *****:********:******  ** :*******:*********

DENV2_NS5       VDTRTQEPKEGTKKLMKITAEWLWKELGKKKTPRMCTREEFTRKVRSNAALGAIFTDENK  418
ZKV_NS5         VDTRVPDPQEGTRQVMNIVSSWLWKELGKRKRPRVCTKEEFINKVRSNAALGAIFEEEKE  419
                ****. :*:***:::*:*.:.********:* **:**:*** .************ :*::

DENV2_NS5       WKSAREAVEDSRFWELVDKERNLHLEGKCETCVYNMMGKREKKLGEFGKAKGSRAIWYMW  478
ZKV_NS5         WKTAVEAVNDPRFWALVDREREHHLRGECHSCVYNMMGKREKKQGEFGKAKGSRAIWYMW  479
                **:* ***:* *** ***:**: **.*:*.:************ ****************

DENV2_NS5       LGARFLEFEALGFLNEDHWFSRENSLSGVEGEGLHKLGYILRDVSKKEGGAMYADDTAGW  538
ZKV_NS5         LGARFLEFEALGFLNEDHWMGRENSGGGVEGLGLQRLGYILEEMNRAPGGKMYADDTAGW  539
                *******************:.**** .**** **::*****.::.:  ** *********

DENV2_NS5       DTRITLEDLKNEEMVTNHMEGEHKKLAEAIFKLTYQNKVVRVQRPTPRG-TVMDIISRRD  597
ZKV_NS5         DTRISKFDLENEALITNQMEEGHRTLALAVIKYTYQNKVVKVLRPAEGGKTVMDIISRQD  599
                ****:  **:** ::**:**  *:.** *::* *******:* **:  * ********:*

DENV2_NS5       QRGSGQVGTYGLNTFTNMEAQLIRQMEGEGVFKSIQHLTITEEIAVQNWLARVGRERLSR  657
ZKV_NS5         QRGSGQVVTYALNTFTNLVVQLIRNMEAEEVLEMQDLWLLRKPEKVTRWLQSNGWDRLKR  659
                ******* **.******: .****:**.* *::  :   : :   * .**   * :**.*

DENV2_NS5       MAISGDDCVVKPLDDRFASALTALNDMGKIRKDIQQWEPSRGWNDWTQVPFCSHHFHELI  717
ZKV_NS5         MAVSGDDCVVKPIDDRFAHALRFLNDMGKVRKDTQEWKPSTGWSNWEEVPFCSHHFNKLY  719
                **:*********:***** **   ******:*** *:*:** **.:* :********::*

DENV2_NS5       MKDGRVLVVPCRNQDELIGRARISQGAGWSLRETACLGKSYAQMWSLMYFHRRDLRLAAN  777
ZKV_NS5         LKDGRSIVVPCRHQDELIGRARVSPGAGWSIRETACLAKSYAQMWQLLYFHRRDLRLMAN  779
                :**** :*****:*********:* *****:******.*******.*:********* **

DENV2_NS5       AICSAVPSHWVPTSRTTWSIHAKHEWMTTEDMLTVWNRVWIQENPWMEDKTPVESWEEIP  837
ZKV_NS5         AICSAVPVDWVPTGRTTWSIHGKGEWMTTEDMLMVWNRVWIEENDHMEDKTPVTKWTDIP  839
                ******* .****.*******.* ********* *******:**  ******* .* :**

DENV2_NS5       YLGKREDQWCGSLIGLTSRATWAKNIQAAINQVRSLIGNE-EYTDYMPSMKRFRREE-EE  895
ZKV_NS5         YLGKREDLWCGSLIGHRPRTTWAENIKDTVNMVRRIIGDEEKYMDYLSTQVRYLGEEGST  899
                ******* *******   *:***:**: ::* ** :**:* :* **: :  *:  ** .

DENV2_NS5       AGVLW        900
ZKV_NS5         PGVL-        903
                 ***
```

Supplementary Figure S1: Sequence alignment for full length NS5s from DENV2 and ZIKV. Sequences were aligned using Clustal Omega. (Sievers, F.; Higgins, D. G., Clustal Omega for making accurate alignments of many protein sequences. Protein Sci. **2018**; 27(1):135-145.)

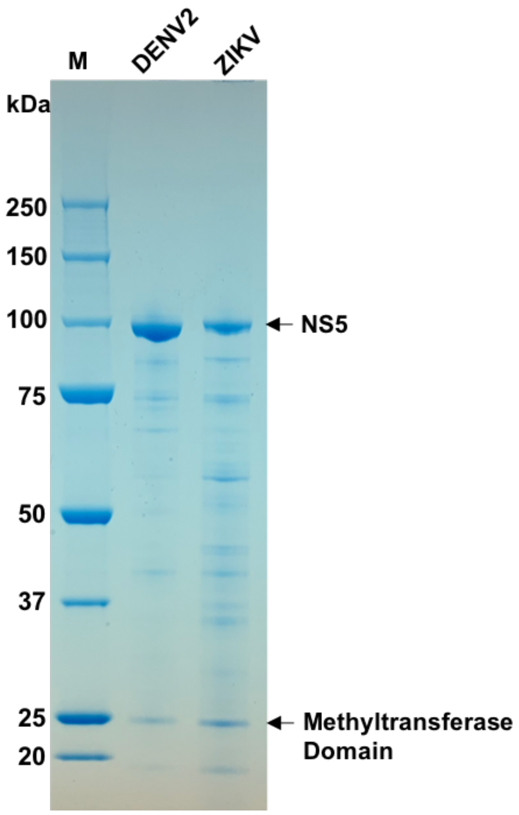

Supplementary Figure S2. Analysis of purified flavivirus NS5 proteins by SDS-PAGE using a NuPAGE™ 4 to 12% Bis-Tris gel and 1x NuPAGE™ MOPS SDS running buffer. Proteins were visualized using colloidal Coomassie staining.

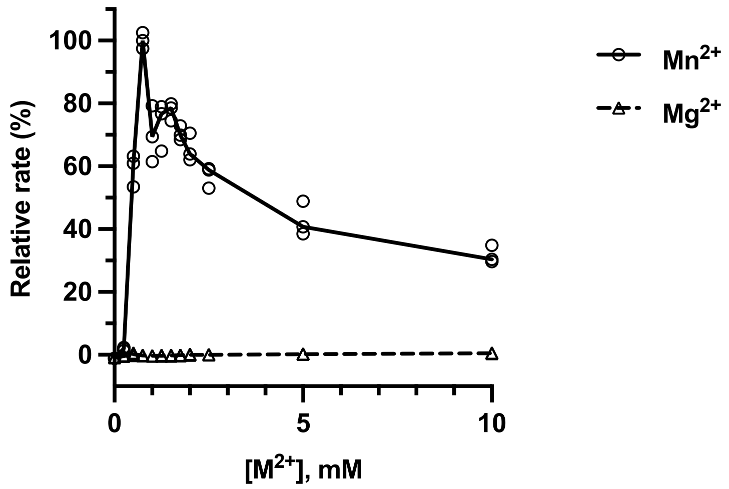

Supplementary Figure S3: Comparison of the RdRp activity of DENV2 NS5 in the presence of Mn^{2+} and Mg^{2+} ions. Reactions contained 200 nM DENV2 NS5, 2.5 μM SYTO 9, 40 μg/ml poly(U), 0 to 10 mM $MnCl_2$ or $MgCl_2$ and were initiated with 0.5 mM ATP. Measured rates in fluorescence units per second are given as a percentage relative to the maximum rate for that experiment. Technical triplicates are shown, connected by a line passing through the median of each set of triplicates.

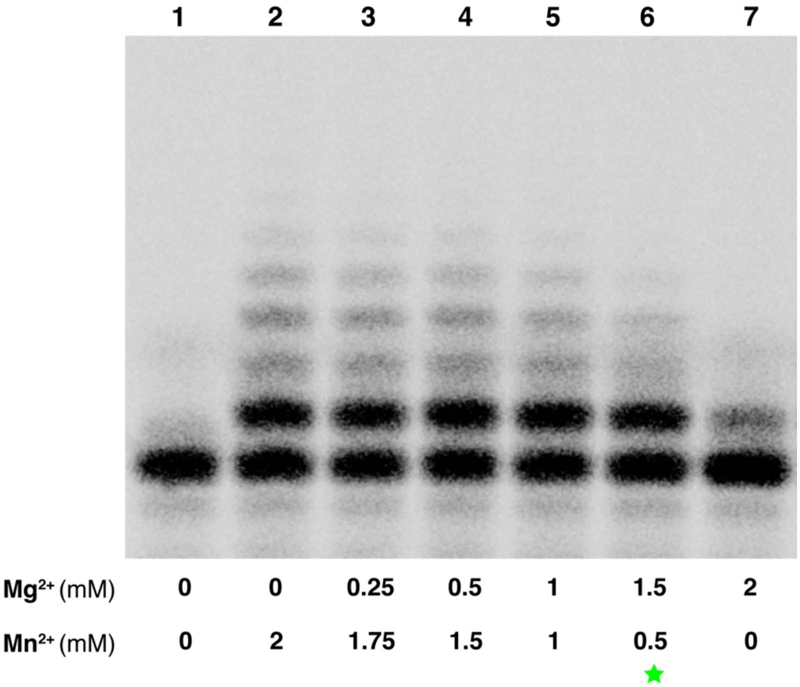

Supplementary Figure S4: Optimization of divalent metal ion concentration for the primer extension assay. Primer extension assay was performed as described in methods using template B (Figure 8A) and in presence of only 10 µM GTP. Lane 1 is the primer by itself. Lane 2-7 are reactions performed using different concentrations of $MgCl_2$ and $MnCl_2$ as indicated. The divalent metal ion concentrations selected for the primer extension assay is highlighted by a green asterisk.

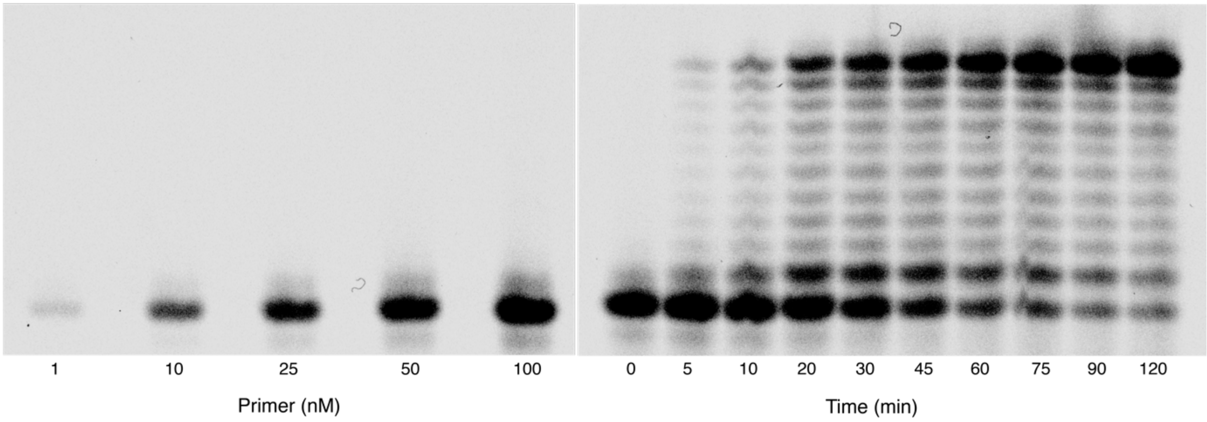

Supplementary Figure S5: Time course of a primer extension reaction with template G (Table 4), analyzed using denaturing polyacrylamide gel electrophoresis, together with band intensity as a function of fluorescently-labeled primer concentration. The leftmost lanes show the band intensity resulting from control reactions lacking NTPs, while incorporating the fluorescently-labeled primer at the indicated concentrations. The rightmost lanes show the product distribution obtained using template G at the indicated times following initiation of the reaction with 5 µM each of ATP, GTP, CTP and UTP.

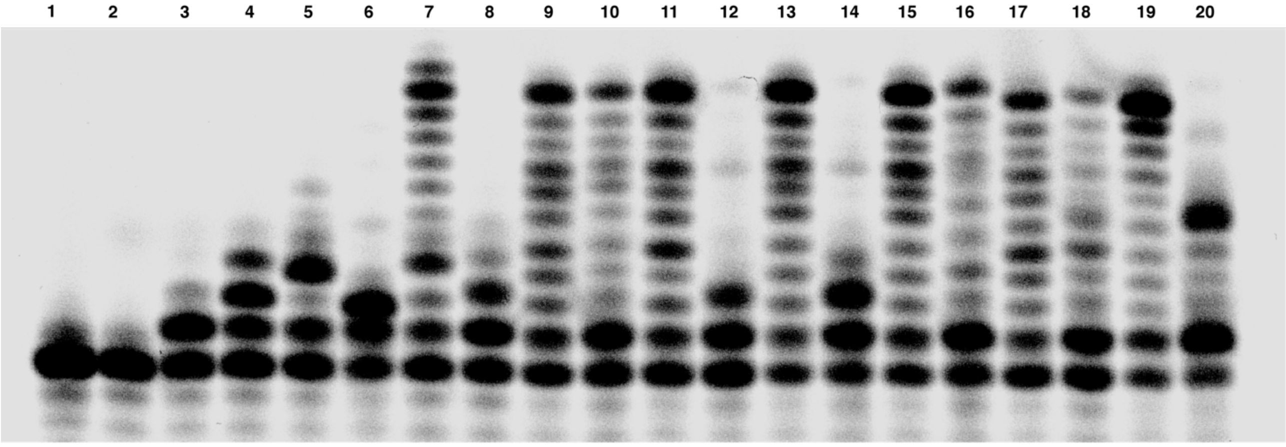

Supplementary Figure S6: Unedited gel image of the results of the primer extension assay analyzed by denaturing polyacrylamide gel electrophoresis. Lane 1 is the primer by itself. Control reactions (lanes 2-6) were performed using template G. Reactions to investigate the effects of Galidesivir incorporation (lanes 7-20) were performed using templates A-F. Full details are given in Figure 8 of the article.

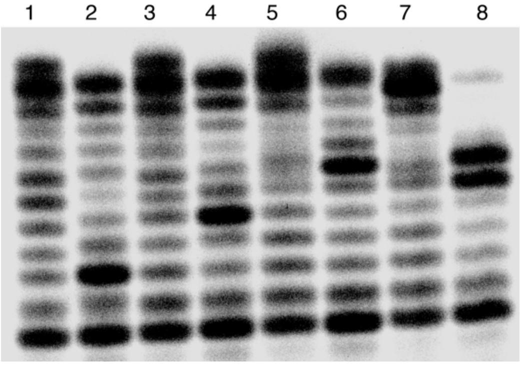

Supplementary Figure S7: Unedited gel image of the results of the primer extension assay using templates H-K analyzed by denaturing polyacrylamide gel electrophoresis. Full details are given in Figure 9 of the article.

```
                                                                                                      DENV2 NS5
    NcoI                     His6-tag            SpeI        spacer         TEV cleavage site      1   2   3       898 899 900        XhoI
     Met Gly Ser Ser His His His His His His Thr Ser Gly Ser Gly Ser Glu Asn Leu Tyr Phe Gln Gly Thr Gly  …  Val Leu Trp ---
ACC ATG GGC AGC AGC CAT CAT CAT CAT CAT CAC ACT AGT GGG AGT GGC AGC GAA AAC CTG TAT TTT CAG GGA ACT GGC  …  GTT CTG TGG TGA CTC GAG

                                                                                                       ZIKV NS5
    NcoI                     His6-tag            SpeI        spacer         TEV cleavage site      1   2   3       901 902 903        XhoI
     Met Gly Ser Ser His His His His His His Thr Ser Gly Ser Gly Ser Glu Asn Leu Tyr Phe Gln Gly Gly Gly  …  Gly Val Leu ---
ACC ATG GGC AGC AGC CAT CAT CAT CAT CAT CAC ACT AGT GGG AGT GGC AGC GAA AAC CTG TAT TTT CAG GGA GGT GGG  …  GGA GTG TTG TGA CTC GAG
```

Supplementary Figure S8. Schematic diagram showing the detail of NS5 gene insertion into the multiple cloning site of vector pET15b(+) (Novagen).

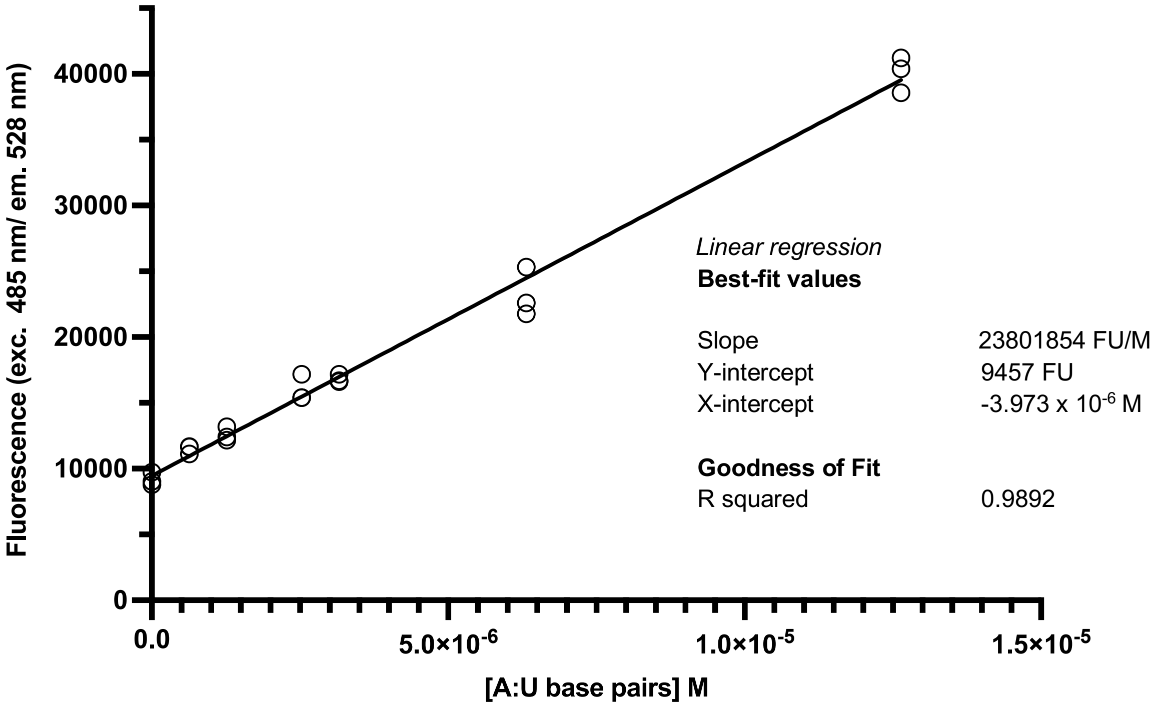

Supplementary Figure S9: Example of a poly(A):poly(U) standard curve in the NS5 RdRp assay conditions (200 nM DENV2 NS5, 20 μM A2 (ApA) primer, 40 μg/ml poly(U), 2.5 mM $MnCl_2$, 3 μM SYTO 9). The poly(A):poly(U) concentration was varied from 0 to 8 μg/ml. This corresponds to an effective A:U base pair concentration of 0 to 12.6 x 10^{-6} M, given a molecular weight of 633.07 $gmol^{-1}$ for each A:U base pair unit.

Section B: Chemical Synthesis of Galidesivir Triphosphate

Experimental Details

Galidesivir,[1] compound **3**,[2] bis(9*H*-fluorenyl-9-methyl) *N*,*N*-diisopropylphosphoramidite,[3] and bis(tributylammonium) pyrophosphate[4] were prepared according to literature procedures. Reactions requiring anhydrous conditions were carried out in flame-dried glassware under a positive pressure of argon in anhydrous solvents, using standard Schlenk techniques. Reaction temperatures above room temperature (22–23 °C) were carried out in heating mantles with an internal temperature probe. Reaction progress was monitored by thin layer chromatography (TLC) on Merck Aluminum-backed silica gel coated TLC plates (60 Å, F254 indicator). TLC plates were visualized by exposure to ultraviolet light (254 nm), and/or staining with ceric ammonium molybdate stain (Hanessian's Stain) or $KMnO_4$ stain. Flash column chromatography was performed with a Büchi Pure C-815 Flash automated flash chromatography system using prepacked FlashPure cartridges containing either silica gel (50 µm irregular) or C18 silica gel (50 µm spherical), and ACS grade solvents. NMR spectra were recorded using a Bruker 500 MHz spectrometer and analyzed using MestReNova software. Data are represented as follows: chemical shift (δ) in parts per million (ppm), multiplicity (s = singlet, d = doublet, dd (doublet of doublets), t = triplet, q = quartet, m = multiplet), coupling constants (*J*) in Hertz (Hz), and integration. High resolution electrospray ionization (ESI) mass spectrometric analysis and liquid chromatography–mass spectrometric analysis (LC-MS) were performed on Waters Q-TOF Premier™ Tandem Mass spectrometer fitted with a Waters 2795 HPLC and analyzed using MassLynx software. LCMS was performed using a ACQUITY UPLC™ BEH C18 column (1.7 µm, 100 × 2.1 mm, 130 Å) and method that used: mobile phase (A: 10 mM ammonium formate in H_2O; B: MeOH), flow rate (0.2 mL/min), temperature (30 °C), and detection method (diode array).

Compound **1:**

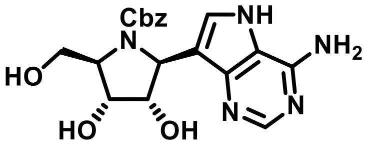

1

To a solution of Galidesivir (115 mg, 0.434 mmol, 1.00 equiv.) in 1,4-dioxane-H_2O (4:1, 2.9 mL) at room temperature was added a solution of *N*-(benzyloxycarbonyloxy)succinimide (120 mg, 0.477 mmol, 1.10 equiv.) in 1,4-dioxane-H_2O (4:1, 1.0 mL). The reaction mixture was stirred for 4 h, then concentrated *in vacuo*. Purification by flash column chromatography (silica gel, 0–20% MeOH-CH_2Cl_2) afforded the title compound (137 mg, 0.343 mmol, 79% yield) as a colorless solid. ^{1}H NMR (500 MHz, MeOD) δ 8.11–8.01 (m, 1H), 7.55–7.21 (m, 3H), 7.17–7.01 (m, 2H), 6.75–6.64 (m, 1H), 5.07–5.01 (m, 1H), 4.83–4.76 (m, 1H, partially obscured by HDO), 4.70 (d, *J* = 12.6 Hz, 1H), 4.64–4.56 (m, 1H), 4.27–4.15 (m, 2H), 3.97 (s, 1H), 3.73 (dd, *J* = 12.3, 2.4 Hz, 1H); ^{13}C NMR (126 MHz, MeOD) δ 157.8, 157.3, 152.1, 150.1, 144.7, 137.9, 137.6, 129.6, 129.5, 129.2, 128.6, 128.1, 116.6, 115.9, 78.1, 77.6, 74.5, 74.1, 69.4, 69.0, 67.9, 67.7, 63.1, 62.8, 61.6, 61.0; HRMS (ESI/QTOF) *m/z* $[M+H]^+$ calculated for $C_{19}H_{22}N_5O_5$ 400.1621, found 400.1612.

Compound **7**:

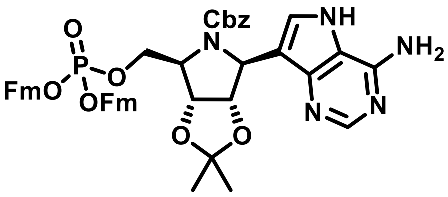

7

To a solution of alcohol **3** (262 mg, 0.596 mmol, 1.00 equiv.) in MeCN (3.0 mL) was added bis(9*H*-fluorenyl-9-methyl) *N*,*N*-diisopropylphosphoramidite (0.67 M in benzene, 0.90 mL, 0.596 mmol, 1.00 equiv.), followed by 1*H*-tetrazole (0.45 M in MeCN, 2.6 mL, 1.2 mmol, 2.0 equiv.). The reaction mixture was stirred at room temperature for 25 min, after which conversion to the phosphite was complete as judged by TLC analysis (2:13:85 Et_3N-MeOH-EtOAc) on deactivated silica. The reaction mixture was cooled to 0 °C, then treated with *tert*-butyl hydroperoxide (70% *w*/*w* in H_2O, 0.21 mL, 1.5 mmol, 2.5 equiv.). The reaction mixture was allowed to warm to room temperature and stirred for 50 min, then diluted with EtOAc (50 mL) and washed with a mixture of sat. aq. $NaHCO_3$ and 1.0 M aq $Na_2S_2O_3$ (1:1, 25 mL). The aqueous layer was extracted with EtOAc (25 mL), then the combined organic phases were washed with sat. aq. $NaHCO_3$ (25 mL), brine (10 mL), dried over anhydrous $MgSO_4$, filtered and concentrated *in vacuo*. Purification by flash column chromatography (silica gel, 0–30% MeOH-EtOAc) afforded the title compound (452 mg, 0.516 mmol, 87% yield) as a colorless foam. ^{1}H NMR (500 MHz, $CDCl_3$) δ 10.81 (s, 1H), 8.31–8.04 (m, 1H), 7.78–7.56 (m, 4H), 7.58–7.09 (m, 18H), 7.03–6.15 (m, 2H), 5.34 (s, 1H), 5.30–5.15 (m, 1H), 5.15–4.87 (m, 3H), 4.49–3.94 (m, 9H), 1.47 (s, 3H), 1.30 (s, 3H); ^{13}C NMR (126 MHz, $CDCl_3$) δ 155.1, 143.0, 142.94, 142.88, 141.4, 140.3, 138.1, 136.3, 128.8, 128.5, 128.1, 128.03, 128.01, 127.6, 127.22, 127.15, 125.1, 121.1, 120.1, 119.8, 114.4, 112.2, 85.0, 83.8, 82.9, 82.1, 69.7, 67.5, 66.5, 64.8, 61.3, 60.5, 47.9, 47.8, 27.4, 25.4; ^{31}P NMR (162 MHz, $CDCl_3$) δ −2.1; HRMS (ESI/QTOF) *m/z* $[M+H]^+$ calculated for $C_{50}H_{47}N_5O_8P$ 876.3157, found 876.3177.

Compound **4**

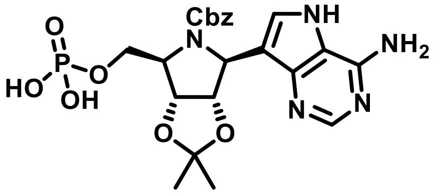

4

From compound **3**:

A solution of compound **3** (115 mg, 0.262 mmol, 1.00 equiv.) in trimethyl phosphate (1.3 mL) was stirred at room temperature under argon for 10 min. This solution was cooled down to 0 °C, then treated dropwise with a solution of $POCl_3$ (0.15 mL, 1.6 mmol, 6.1 equiv.) in trimethyl phosphate (1.3 mL). The

reaction mixture was stirred at 0 °C for 1 h, then at room temperature for 2 h. Consumption of the starting material was confirmed by TLC (normal phase silica gel using a mixture of *i*-PrOH-H_2O-conc. aqueous NH_3 (4:2:4)). The reaction was quenched by addition of 2 M aqueous triethylammonium bicarbonate buffer (pH 7) (4.0 mL), concentrated *in vacuo* to a white solid, and purified by by flash column chromatography (C18 reversed-phase column, 0–50% H_2O with 0.1% formic acid/MeOH) to afford the title compound (49.0 mg, 0.094 mmol, 36% yield) as a colorless powder. Full characterization was performed using the internal salt of compound **3**.

From compound **7**:

To a solution of compound **7** (284 mg, 0.324 mmol, 1.00 equiv.) in DMF (3.2 mL) at room temperature was added piperidine (0.32 mL, 3.2 mmol, 10 equiv.). The reaction mixture was stirred for 3 h, then concentrated *in vacuo*. The residue was dissolved in MeOH (10 mL), then filtered to remove insoluble fulvene byproducts. The filtrate was concentrated *in vacuo*, then the solid obtained was washed with Et_2O (3 × 5 mL) to further remove fulvene byproducts. The solid was purified by flash column chromatography (C18 reversed-phase column, 10–80% H_2O-MeOH) to afford the title compound (181 mg, 0.282 mmol, 87% yield as a piperidinium salt) as a colorless solid.

^{1}H NMR (500 MHz, MeOD) δ 8.42–7.71 (m, 2H), 7.46–6.53 (m, 5H), 5.17–4.93 (m, 3H), 4.85–4.52 (m, 2H), 4.47–4.04 (m, 3H), 1.51 (s, 3H), 1.30 (s, 3H); ^{13}C NMR (126 MHz, MeOD) δ 156.4, 152.6, 145.3, 136.9, 132.1, 129.2, 128.8, 115.0, 113.2, 88.5, 86.7, 83.6, 82.9, 68.5, 66.0, 65.8, 62.0, 61.4, 28.0, 25.8; ^{31}P NMR (202 MHz, MeOD) δ 0.6. HRMS (ESI/QTOF) *m/z* [M−H]$^-$ calculated for $C_{22}H_{25}N_5O_8P$ 518.1446, found 518.1445.

Compound **5**

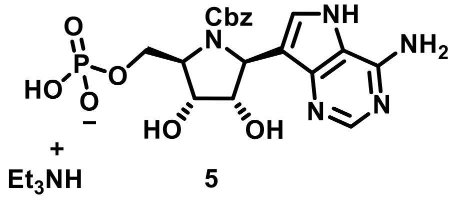

5

To a suspension of compound **4** (piperidinium salt, 240 mg, 0.397 mmol) in CH_2Cl_2 (4.0 mL) was added TFA (4.0 mL) at room temperature. The reaction was stirred for 18 h, then concentrated *in vacuo*. The residue thus obtained was purified by flash column chromatography (C18 reversed-phase column, 10–40% H_2O-MeOH) then treated with DOWEX® 50W X8 Et_3NH^+ to afford the title compound (225 mg, 0.388 mmol, 97% yield as the triethylammonium salt) as a colorless solid. ^{1}H NMR (500 MHz, D_2O) δ 8.22–7.74 (m, 2H), 7.42–6.89 (m, 3H), 6.64–6.39 (m, 2H), 4.78–4.61 (m, 3H), 4.54–4.39 (m, 2H), 4.39–4.25 (m, 1H), 4.25–4.15 (m, 1H), 4.15–4.10 (m, 1H); ^{13}C NMR (126 MHz, D_2O) δ 156.5, 149.4, 143.5, 137.7, 134.6, 130.8, 128.3, 127.8, 113.8, 111.8, 77.7, 72.9, 67.6, 65.9, 63.4, 55.8; ^{31}P NMR (202 MHz, D_2O) δ 2.3. HRMS (ESI/QTOF) *m/z* [M−H]$^-$ calculated for $C_{19}H_{21}N_5O_8P$ 478.1133, found 478.1136.

Compound **2**

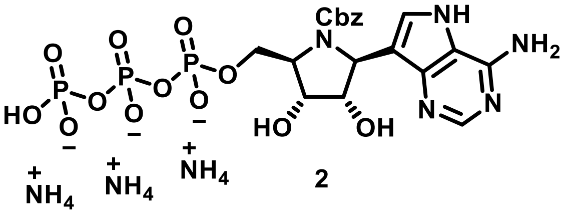

From compound **1**:

To a stirred solution of compound **1** (149 mg, 0.373 mmol, 1.0 equiv.) in trimethyl phosphate (1.5 mL) at 0 °C was added a solution of $POCl_3$ (0.14 mL, 1.5 mmol, 4.0 equiv.) in trimethyl phosphate (1.5 mL) dropwise. The reaction mixture was stirred at 0 °C for 3 h, after which conversion to the monophosphate was confirmed by TLC analysis (normal phase silica gel using a mixture of *i*-PrOH-H_2O-conc. aqueous NH_3 (5:1:3)). A solution of tributylammonium pyrophosphate (620 mg, 1.13 mmol, 3.0 equiv.) and tributylamine (0.9 mL, 4 mmol, 10 equiv.) in MeCN (2 mL) was added and the reaction mixture was stirred for 2 h, then quenched by addition of 2 M aqueous triethylammonium bicarbonate buffer (pH 7) (2.0 mL). The reaction mixture was washed with CH_2Cl_2 (4 ×), then the aqueous phase was concentrated *in vacuo*. The crude residue was purified by ion pair chromatography (C18 reversed-phase column, 0–50% mobile phases A/B; mobile phase A composed of deionized H_2O with 10 mM tributylamine and 30 mM acetic acid; mobile phase B composed of MeOH with 15 mM tributylamine), then treated with DOWEX® 50W X8 NH_4^+ form to afford the title compound (85 mg, 0.12 mmol, 33% yield as the tri(ammonium) salt) as a white powder contaminated with phosphate impurities.

From compound **5**:

CDI (46 mg, 0.28 mmol, 5.9 equiv.) was added to a solution of compound **5** (27 mg, 47 µmol, 1.0 equiv.) in anhydrous DMF (0.3 mL) at room temperature and the reaction mixture was stirred under argon for 3 h. Consumption of the starting material was monitored by TLC (normal phase silica gel using a mixture of *i*-PrOH-H_2O-conc. aqueous NH_3 (6:1:3)). A 5% triethylamine solution in MeOH-H_2O (1:1, 1 mL) was added to the reaction mixture and stirred at room temperature for 4 h. Hydrolysis of the 2′,3′-cyclic carbonate was monitored by LCMS, then the reaction mixture was concentrated *in vacuo*. The residue thus obtained was dissolved in anhydrous DMF (0.3 mL), to which was added a solution of tributylammonium pyrophosphate (78 mg, 0.14 mmol, 3.1 equiv.) in anhydrous DMF (0.3 mL) at room temperature. The reaction mixture was stirred for 20 h, after which conversion of the monophosphate to triphosphate was confirmed by TLC (normal phase silica gel using a mixture of *i*-PrOH-H_2O-conc. aqueous NH_3 (4:2:4)). The reaction mixture was diluted with 2 M aqueous triethylammonium bicarbonate buffer (pH 7) (2.0 mL) and concentrated *in vacuo*. The crude residue was purified by ion pair chromatography (C18 reversed-phase column, 0–50% mobile phases A/B; mobile phase A composed of deionized H_2O with 10 mM tributylamine and 30 mM acetic acid; mobile phase B composed of MeOH with 15 mM tributylamine), then treated with DOWEX® 50W X8 NH_4^+ form to afford the title compound (18 mg, 26 µmol, 56% yield as the tri(ammonium) salt) as a colorless powder.

^{1}H NMR (500 MHz, D_2O) δ 8.10–7.84 (m, 2H), 7.56–6.97 (m, 4H), 6.97–6.69 (m, 2H), 5.16 (d, *J* = 11.4 Hz, 1H), 4.77–4.60 (m, 3H), 4.57–4.52 (m, 1H), 4.45–4.31 (m, 2H), 4.18–4.14 (m, 1H); ^{13}C NMR (126 MHz, D_2O) δ 156.7, 150.9, 144.0, 134.9, 134.8, 131.4, 131.3, 128.5, 128.2, 127.9, 111.9, 111.8, 77.2, 72.4, 67.9, 65.4, 64.8, 64.7, 55.4. ^{31}P NMR (202 MHz, D_2O) δ −10.4 (d, *J* = 19.1 Hz), −11.4 (d, *J* = 18.9 Hz), −22.9 (t, *J* = 18.7 Hz). HRMS (ESI/TOF) *m/z* $[M+H]^+$ calculated for $C_{19}H_{25}N_5O_{14}P_3$ 640.0605, found 640.0601.

Galidesivir triphosphate

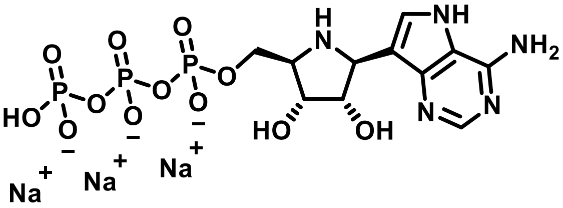

A solution of compound **2** (20 mg, 29 µmol) in deionized H_2O (2 mL) was degassed with argon, then stirred with palladium on carbon (10% *w/w*, 20 mg) under a hydrogen atmosphere (balloon) at room temperature for 1 h. Cbz deprotection was monitored by TLC (normal phase silica gel using a mixture of *i*-PrOH-H_2O-conc. aqueous NH_3 (5:2:3)). The reaction mixture was filtered through a pad of Celite®, washing with then deionized H_2O, then the filtrate was lyophilized. The residue thus obtained was purified by ion pair chromatography (C18 reversed-phase column, 0–30% mobile phases A/B; mobile phase A composed of deionized H_2O with 10 mM tributylamine and 30 mM acetic acid; mobile phase B composed of MeOH with 15 mM tributylamine), then treated with DOWEX® 50W X8 Na^+ form to afford the title compound (11.0 mg, 19.3 µmol, 66% yield assuming trisodium salt form) as a white powder. Full characterization was performed using the bis(tributylammonium) salt of compound **11**. ^{1}H NMR (500 MHz, D_2O) δ 8.31 (s, 1H), 8.05 (s, 1H), 4.99 (d, *J* = 8.6 Hz, 1H), 4.77–4.74 (m, 1H), 4.60–4.49 (m, 2H), 4.49–4.38 (m, 1H), 4.15–4.06 (m, 1H), 3.22–3.01 (m, 12H), 1.78–1.66 (m, 12H), 1.43 (m, 12H), 0.99 (t, *J* = 7.4 Hz, 18H); ^{13}C NMR (126 MHz, D_2O) δ 150.4, 150.3, 146.7, 146.6, 139.2, 139.1, 131.8, 113.0, 112.9, 104.9, 73.4, 70.1, 63.8, 63.7, 63.0, 55.7, 52.7, 47.3, 27.5, 25.2, 19.3, 19.2, 12.8; ^{31}P NMR (202 MHz, D_2O) δ −8.7 (d, *J* = 20.2 Hz), −11.6 (d, *J* = 19.6 Hz), −22.4 (t, *J* = 17.2 Hz). HRMS (ESI-/QTOF) *m/z* $[M-H]^-$ calculated for $C_{11}H_{17}N_5O_{12}P_3$ 504.0092, found 504.0093. The purity of the triphosphate was analyzed by LCMS using diode array as the detection method.

1. Evans, G. B.; Furneaux, R. H.; Gainsford, G. J.; Schramm, V. L.; Tyler, P. C., Synthesis of Transition State Analogue Inhibitors for Purine Nucleoside Phosphorylase and N-Riboside Hydrolases. *Tetrahedron* **2000,** *56* (19), 3053–3062.

2. Sparrow, K. J.; Shrestha, R.; Wood, J. M.; Clinch, K.; Hurst, B. L.; Wang, H.; Gowen, B. B.; Julander, J. G.; Tarbet, E. B.; McSweeney, A. M.; Ward, V. K.; Evans, G. B.; Harris, L. D., An Isomer of Galidesivir That Potently Inhibits Influenza Viruses and Members of the *Bunyavirales* Order. ACS Med Chem Lett. **2023**, 14(4), 506-513.

3. Caron, J.; Lepeltier, E.; Reddy, H.; Lepêtre-Mouelhi, S.; Wack, S.; Bourgaux, C.; Couvreur, P.; Desmaële, D., Squalenoyl Gemcitabine Monophosphate: Synthesis, Characterisation of Nanoassemblies and Biological Evaluation. *Eur. J. Org. Chem.* **2011**, *14*, 2615−2628.

4. Thillier, Y.; Sallamand, C.; Baraguey, C.; Vasseur, J.-J.; Debart, F., Solid-Phase Synthesis of Oligonucleotide 5'-(α-P-Thio)triphosphates and 5'-(α-P-Thio)(β,γ-methylene)triphosphates. *European J. Org. Chem.* **2015,** *2015* (2), 302–308.

Compound **1**

^{1}H NMR (500 MHz, MeOD)

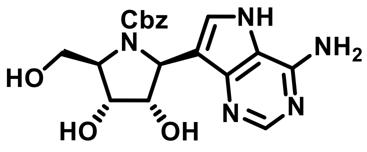

1

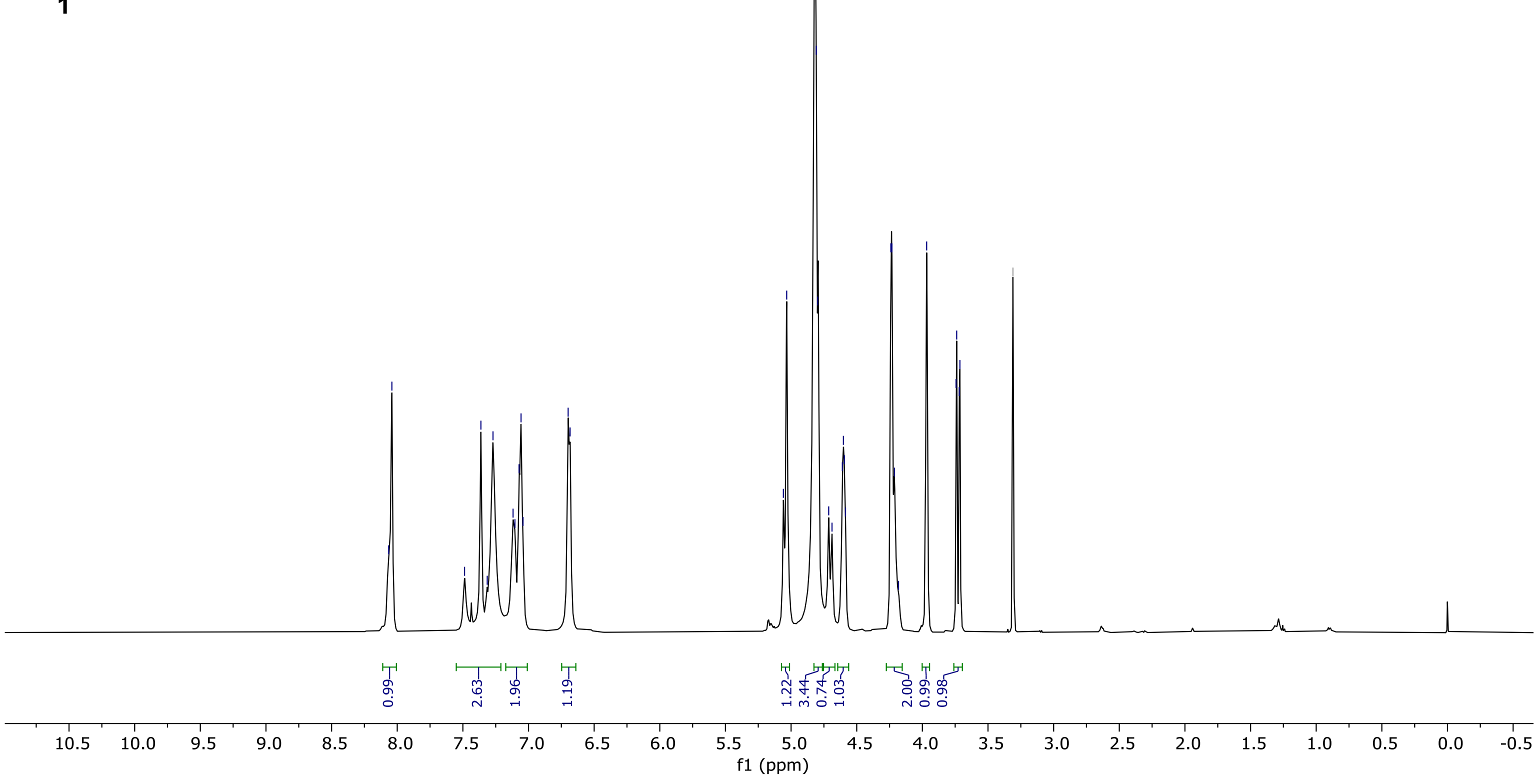

^{13}C NMR (126 MHz, MeOD)

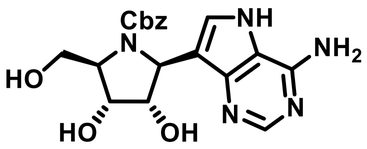

1

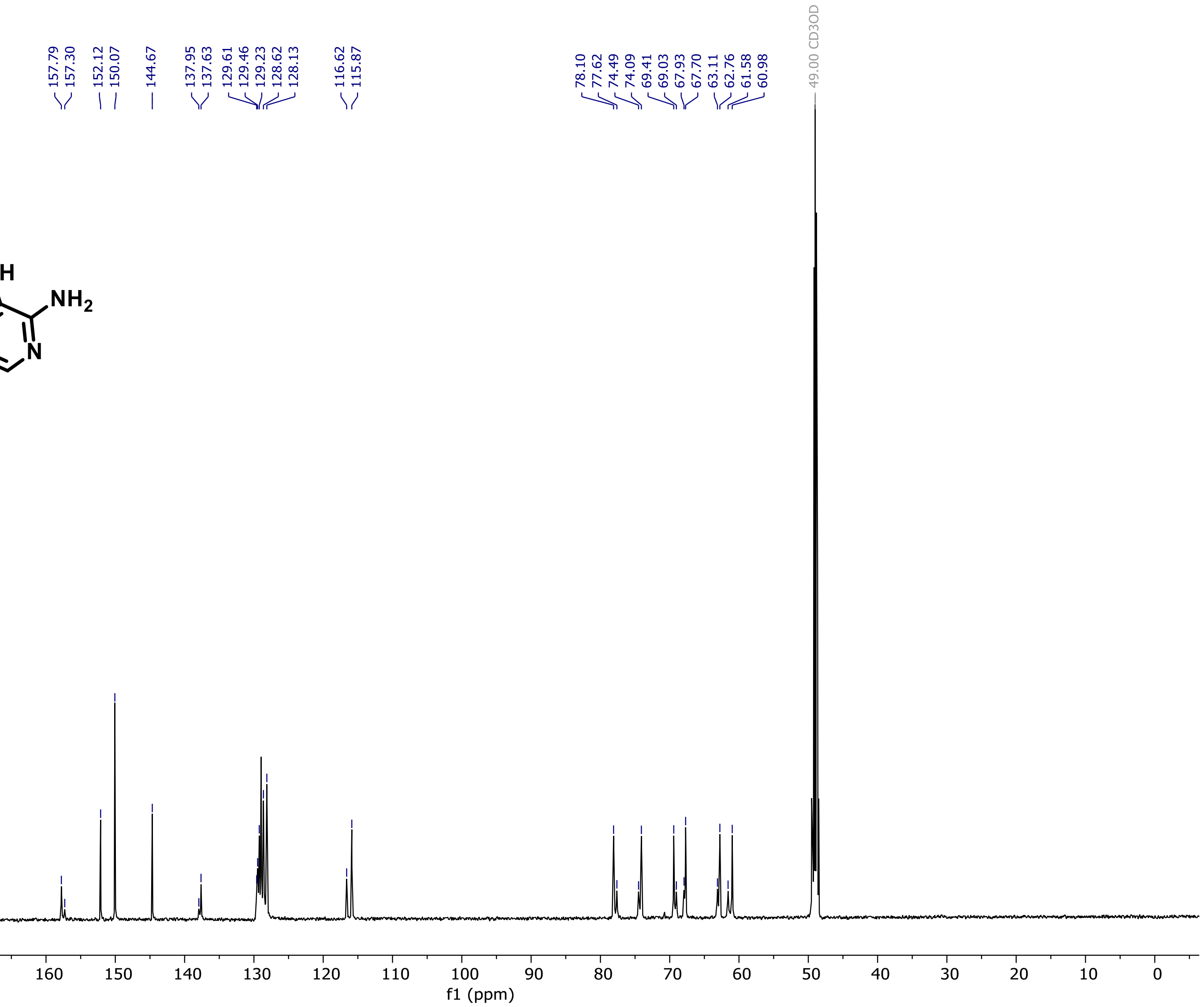

HRMS (ESI/TOF)

Elemental Composition Report

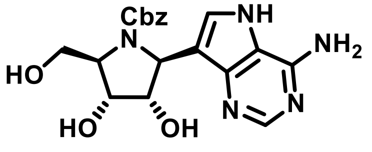

1

Page 1

Single Mass Analysis

Tolerance = 5.0 mDa / DBE: min = -1.5, max = 150.0
Element prediction: Off
Number of isotope peaks used for i-FIT = 3

Monoisotopic Mass, Even Electron Ions
132 formula(e) evaluated with 4 results within limits (up to 100 closest results for each mass)
Elements Used:
C: 0-80 H: 0-100 N: 5-5 O: 0-20 Na: 0-1

RSh-AV20-051b 23 (0.248)

20-Jul-2020
1: TOF MS ES+
3.85e+006

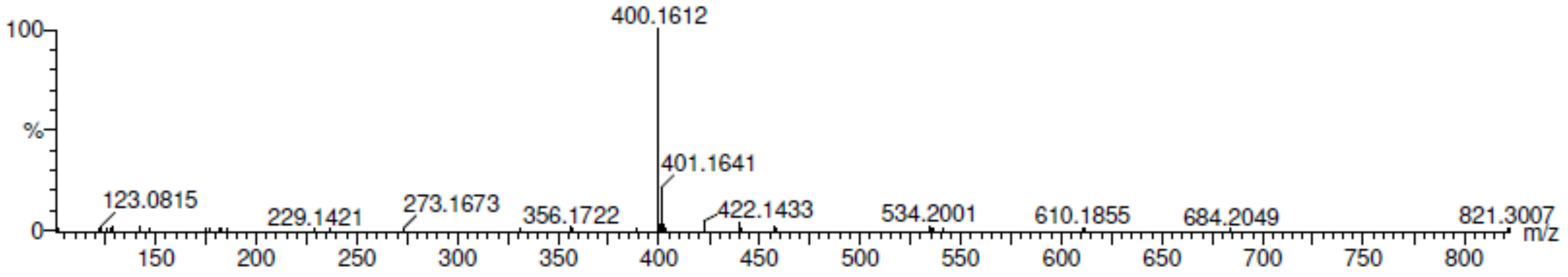

Minimum:				-1.5				
Maximum:		5.0	5.0	150.0				

Mass	Calc. Mass	mDa	PPM	DBE	i-FIT	Norm	Conf(%)	Formula
400.1612	400.1621	-0.9	-2.2	11.5	51.1	1.703	18.21	C19 H22 N5 O5
	400.1597	1.5	3.7	8.5	49.6	0.202	81.70	C17 H23 N5 O5 Na
	400.1656	-4.4	-11.0	-0.5	56.7	7.273	0.07	C10 H27 N5 O10 Na
	400.1562	5.0	12.5	20.5	57.7	8.298	0.02	C26 H18 N5

Compound **7**

^{1}H NMR (500 MHz, $CDCl_3$)

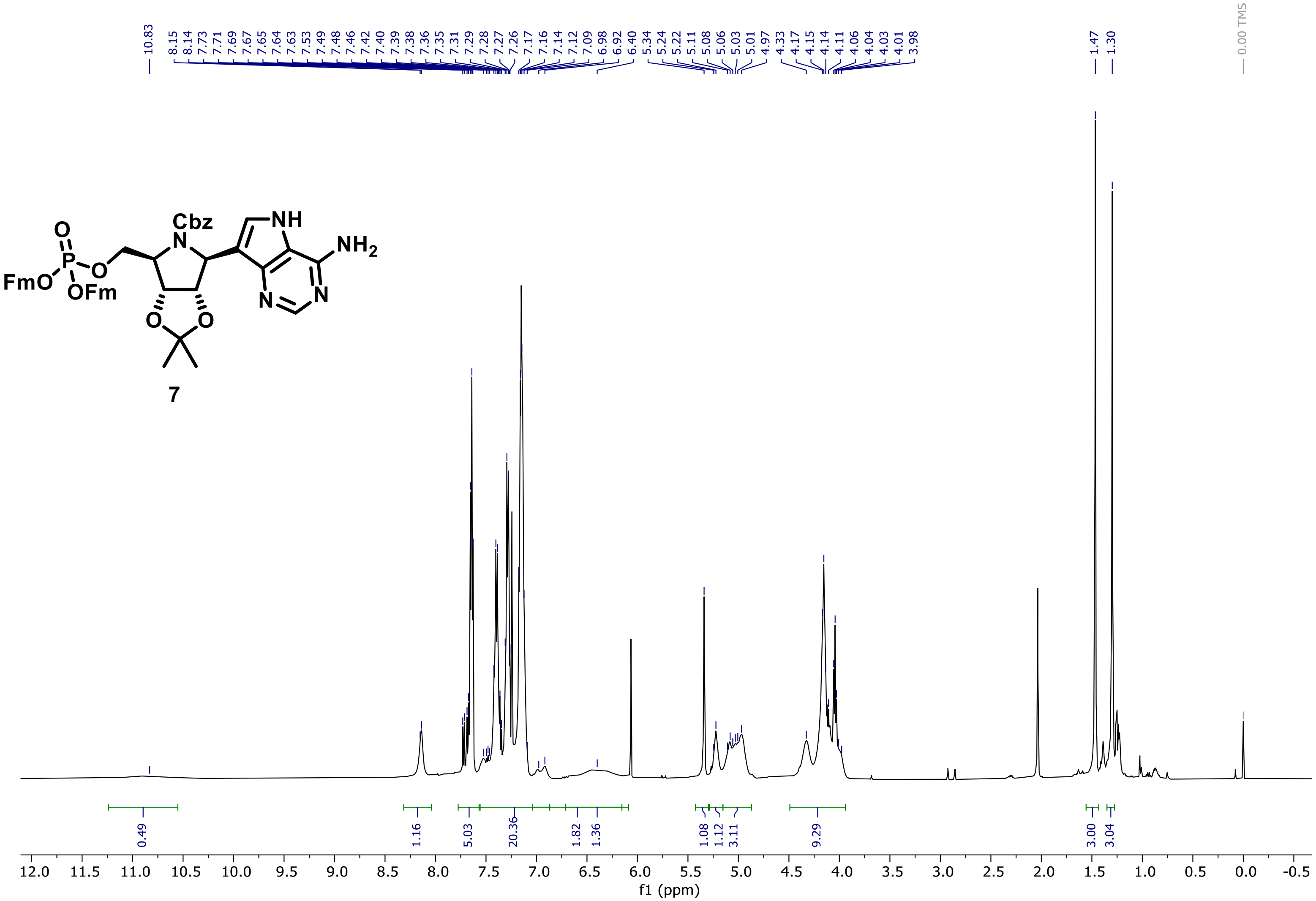

^{13}C NMR (126 MHz, $CDCl_3$)

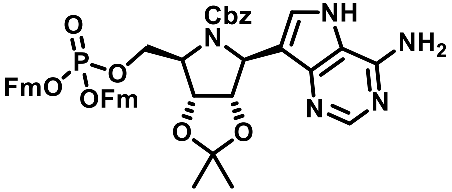

7

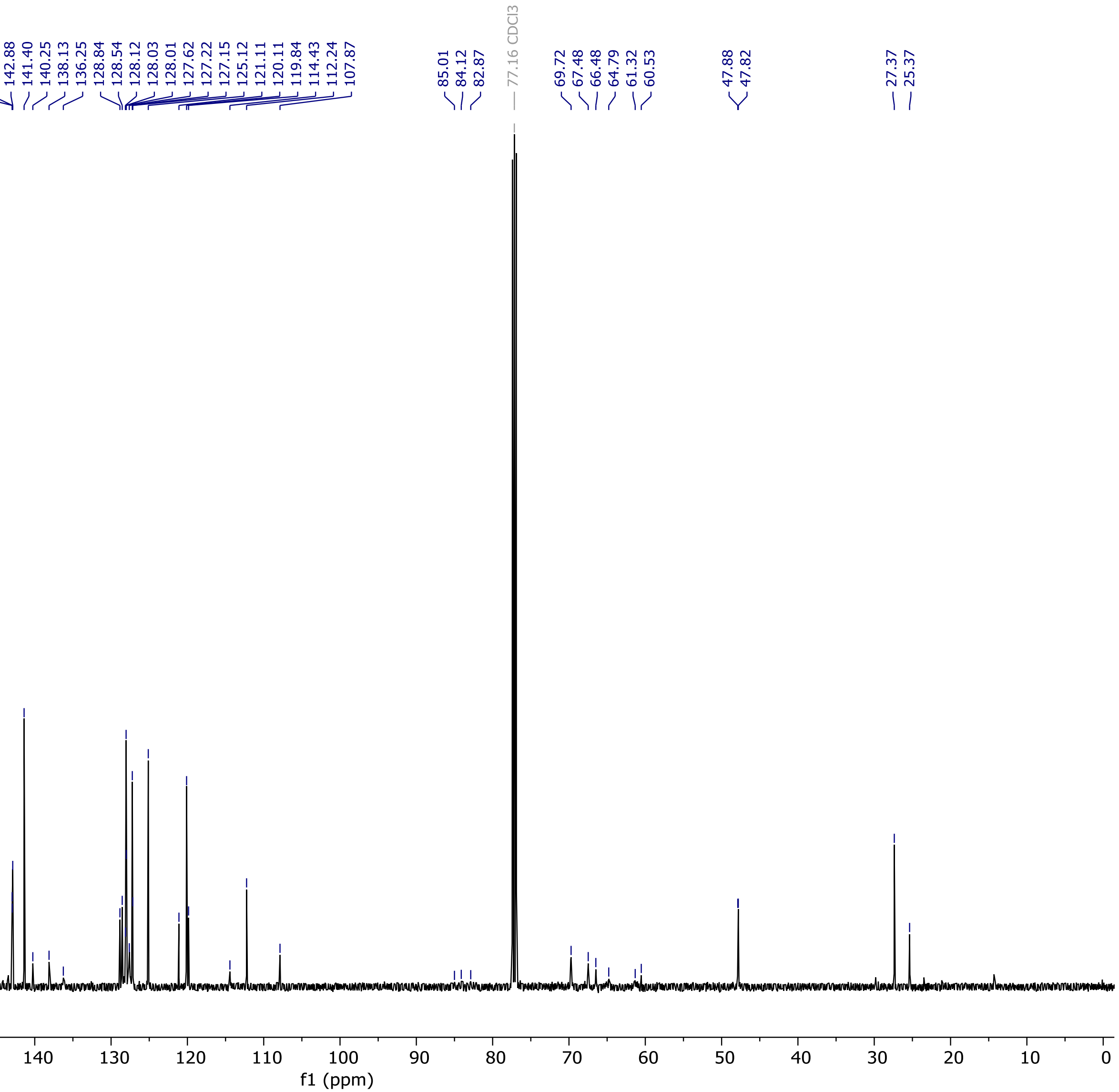

^{31}P NMR (202 MHz, $CDCl_3$)

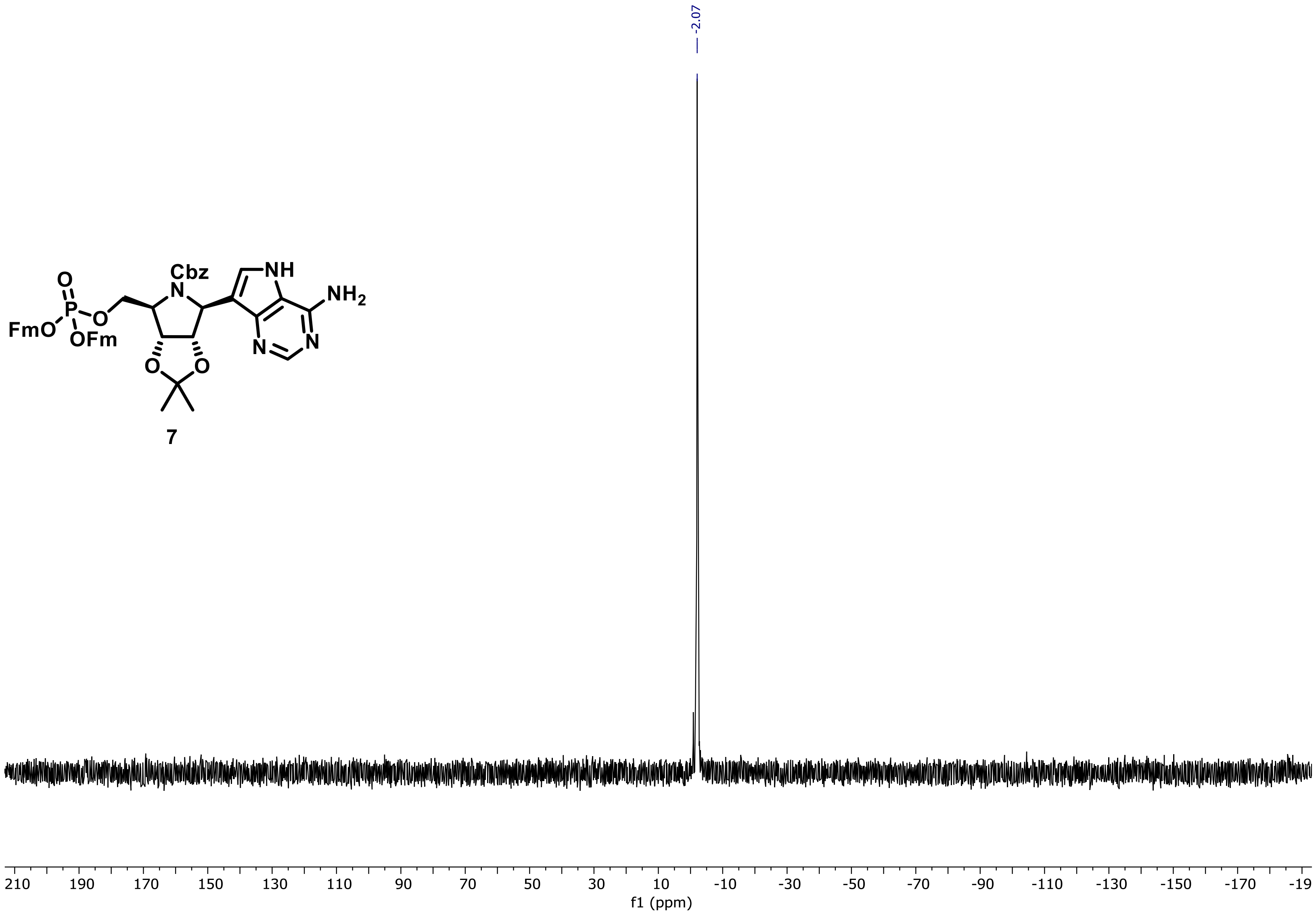

7

HRMS (ESI/QTOF)

Sample Information

**Name**	JMW-AV23-042	**Data File Path**	Z:\Data\Callaghan\JMW-AV23-042_0010.d
**Sample ID**		**Acq. Time (Local)**	13/04/2023 2:41:01 pm (UTC+12:00)
**Instrument**	Instrument 1	**Method Path (Acq)**	D:\MassHunter\Methods\no_column_pos.m
**MS Type**	QTOF	**Version (Acq SW)**	6200 series TOF/6500 series Q-TOF 10.1 (48.0)
**Inj. Vol. (ul)**	1	**IRM Status**	Some ions missed
**Position**	Vial 7	**Method Path (DA)**	C:\Users\masspec\OneDrive - Victoria University of Wellington - STAFF\Documents\jan.m
**Plate Pos.**		**Target Source Path**	
**Operator**		**Result Summary**	1 qualified (1 targets)

Compound Summary

Cpd	Name	Formula	CAS	RT	Mass	Mass (Tgt)	Diff (Tgt, ppm)	Score	Algorithm
1		C50 H46 N5 O8 P		0.210	875.3105	875.3084	2.38	96.28	FBF

Compound Details

Cpd. 1: C50 H46 N5 O8 P

Name	Formula	RT	RI	Mass	Diff (Tgt, ppm)	CAS	ID Source	Score	Algorithm
	C50 H46 N5 O8 P	0.210		875.3105	2.38		FBF	96.28	FBF

Species	m/z	Score (Tgt)	Score (Lib)	Score (DB)	Score (MFG)	Score (RT)
(M+H)+ (M+Na)+	876.3177 898.2976	96.28				

Compound Chromatograms (overlaid)

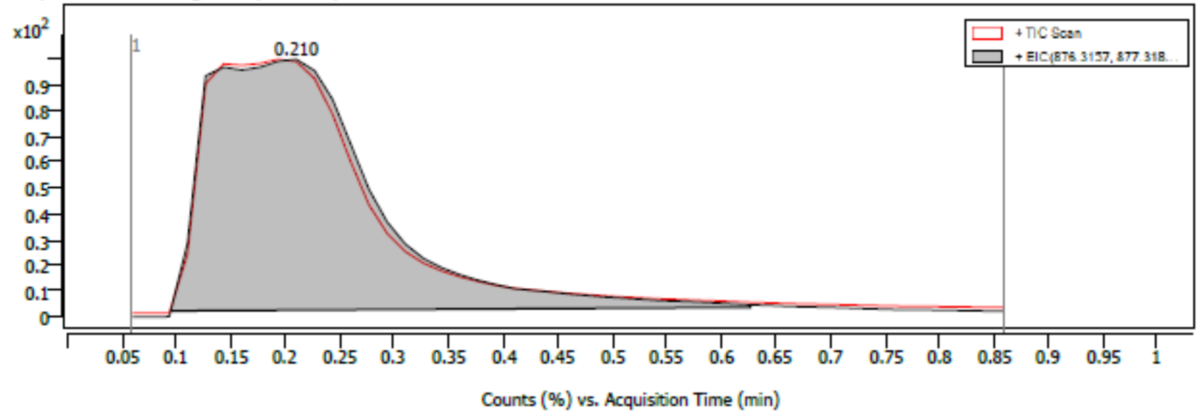

Structure

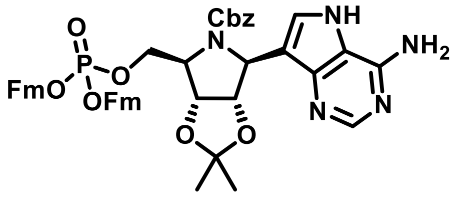

7

Compound **4**

^{1}H NMR (500 MHz, MeOD)

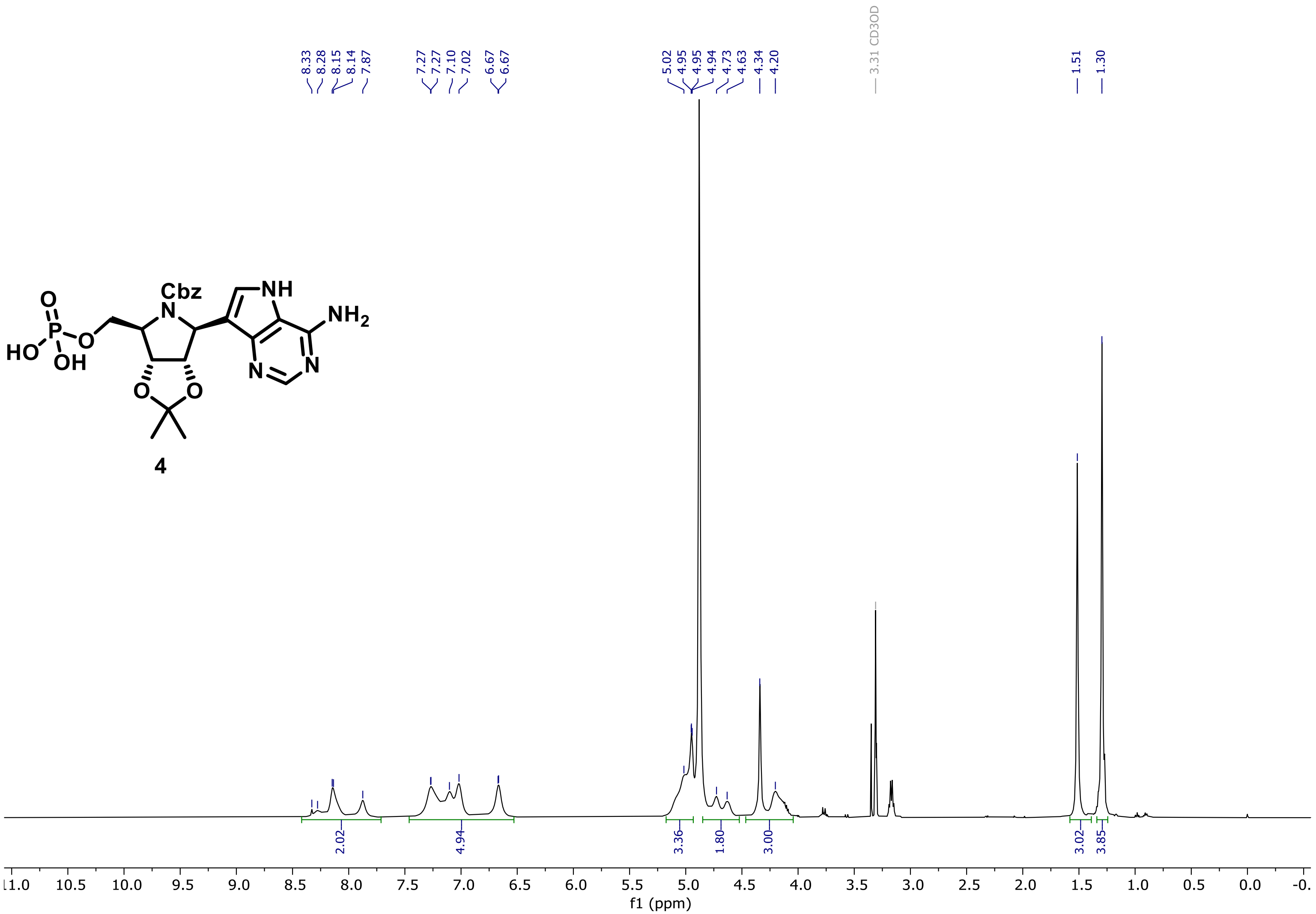

^{13}C NMR (126 MHz, MeOD)

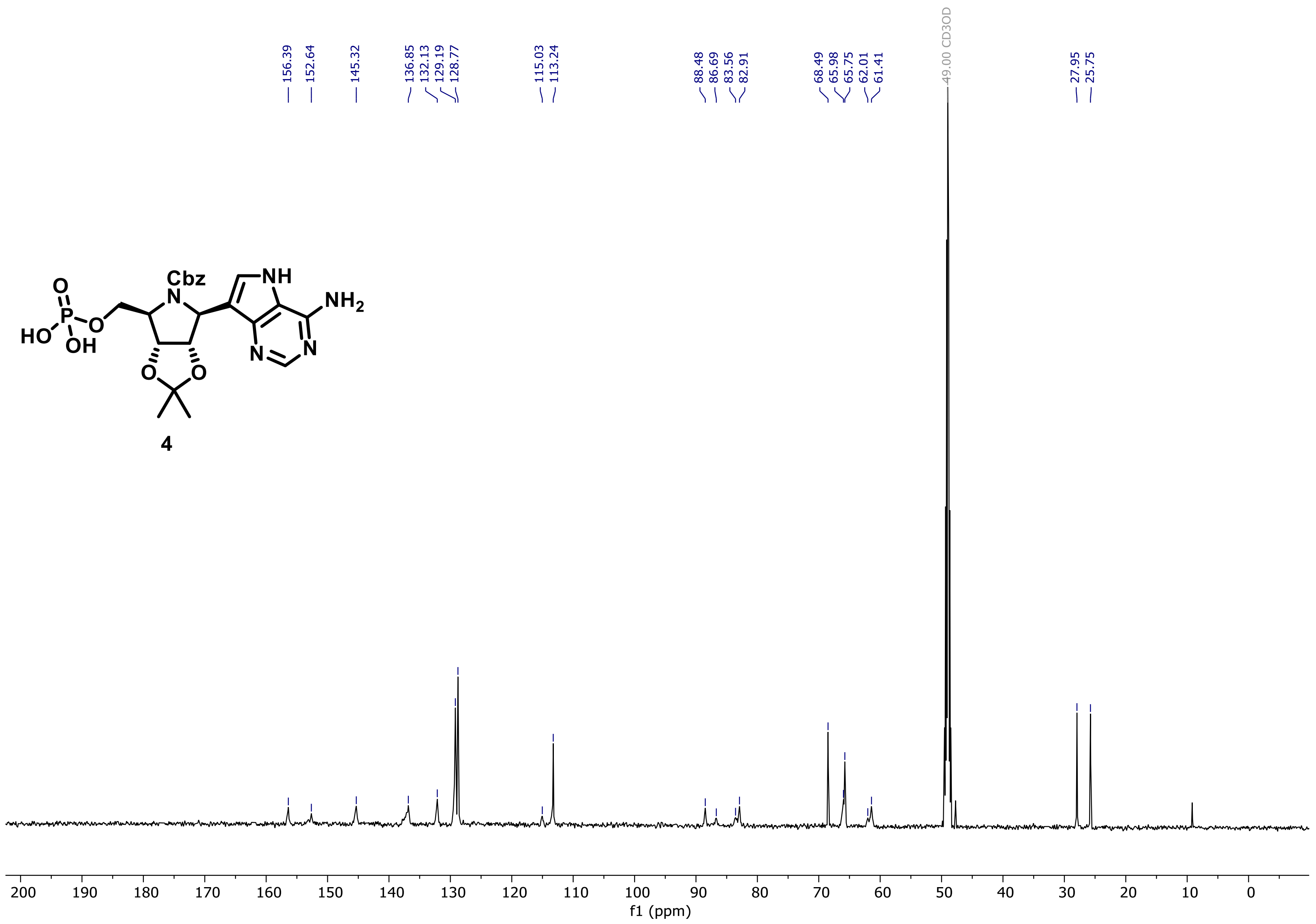

4

^{31}P NMR (202 MHz, MeOD)

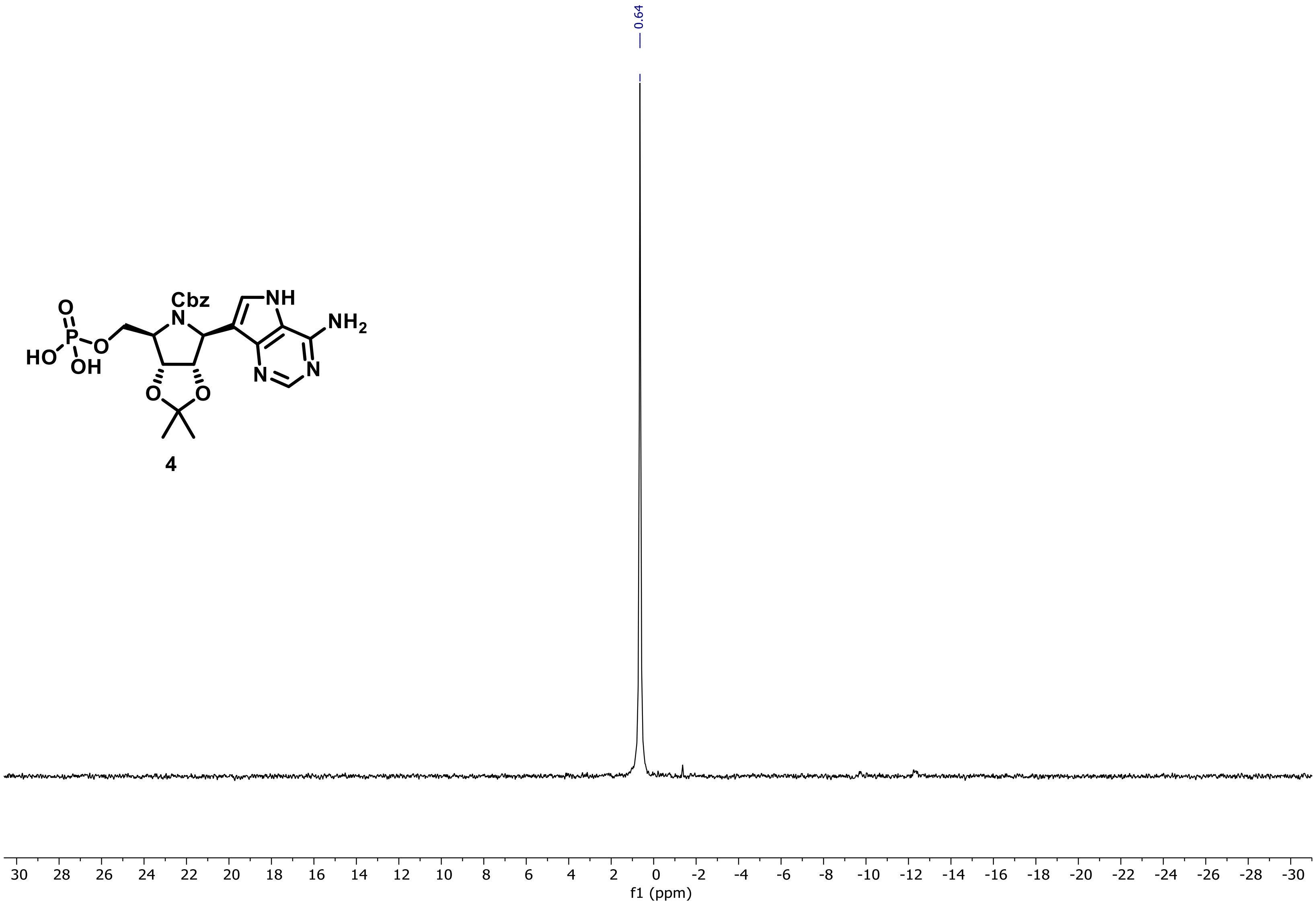

HRMS (ESI/QTOF)

Elemental Composition Report

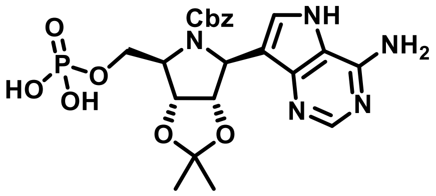

4

Single Mass Analysis

Tolerance = 5.0 mDa / DBE: min = -1.5, max = 150.0
Element prediction: Off
Number of isotope peaks used for i-FIT = 3

Monoisotopic Mass, Even Electron Ions
194 formula(e) evaluated with 3 results within limits (up to 100 closest results for each mass)
Elements Used:
C: 0-120 H: 0-120 N: 5-5 O: 0-40 Na: 0-1 P: 1-1

RSh-AV20-067c- 18 (0.171) AM2 (Ar,22000.0,248.96,0.00); ABS; Cm (15:20)

04-Aug-2020
TOF MS ES-
2.16e+007

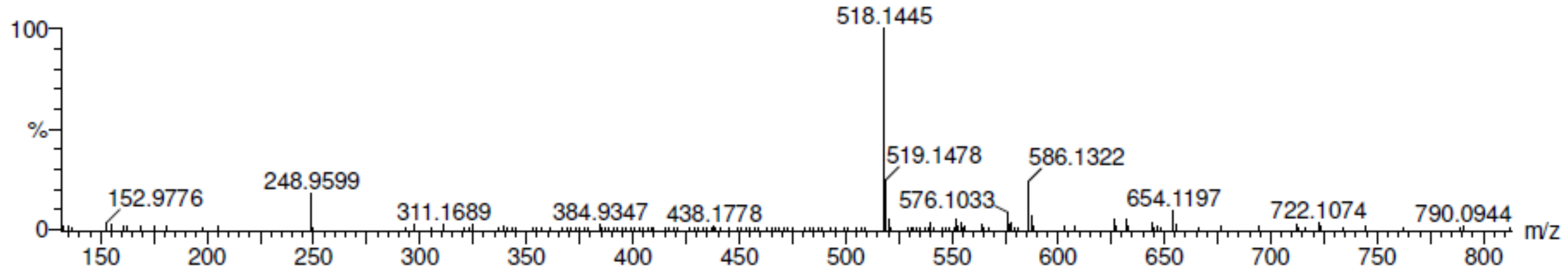

Mass	Calc. Mass	mDa	PPM	DBE	i-FIT	Norm	Conf(%)	Formula
Minimum:				-1.5				
Maximum:		5.0	5.0	150.0				
518.1445	518.1441	0.4	0.8	13.5	59.3	6.570	0.14	C22 H25 N5 O8 P
	518.1417	2.8	5.4	10.5	52.8	0.001	99.86	C20 H26 N5 O8 Na P
	518.1475	-3.0	-5.8	1.5	65.1	12.300	0.00	C13 H30 N5 O13 Na P

Compound **5**

^{1}H NMR (500 MHz, D_2O)

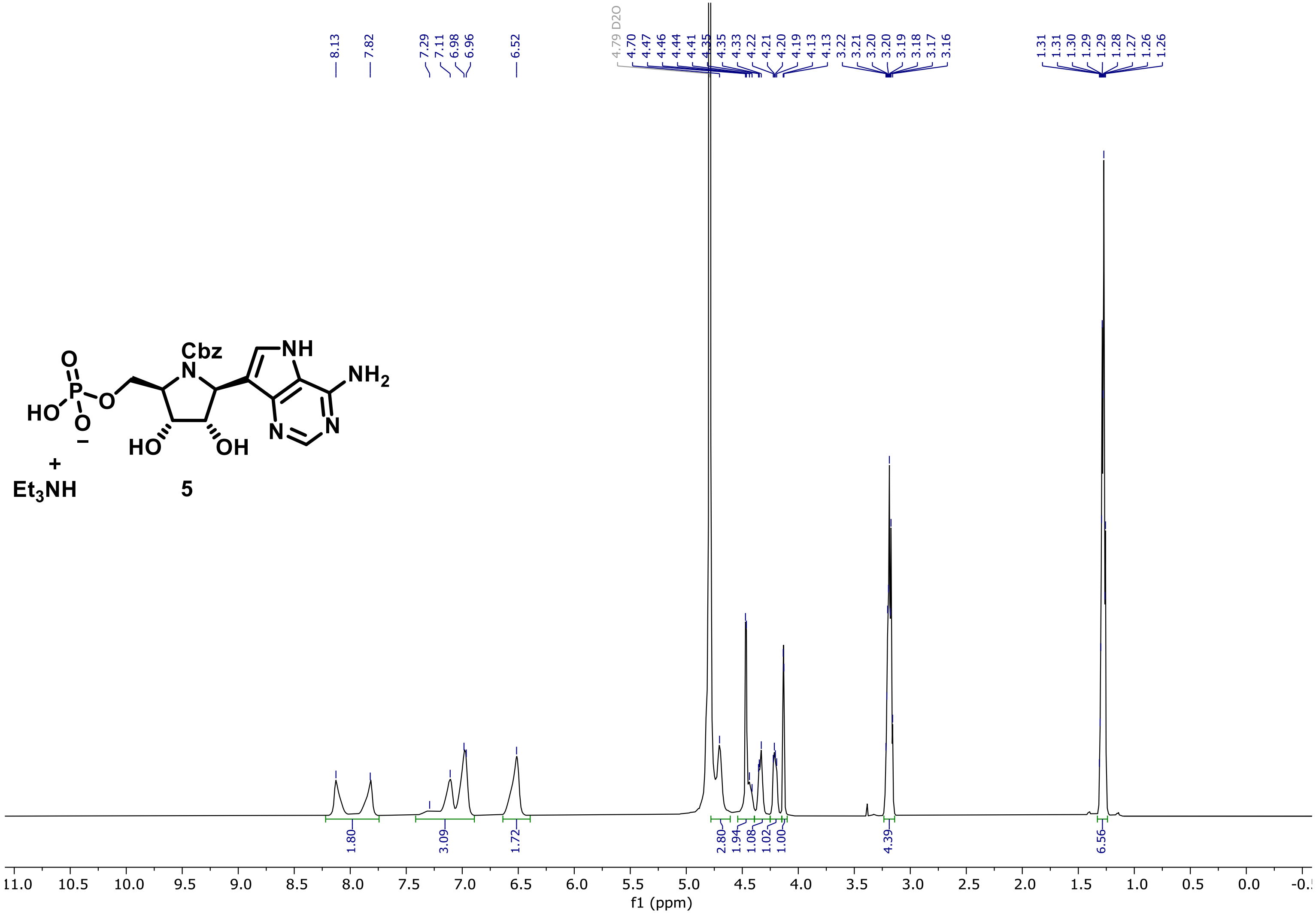

5

^{13}C NMR (126 MHz, D_2O)

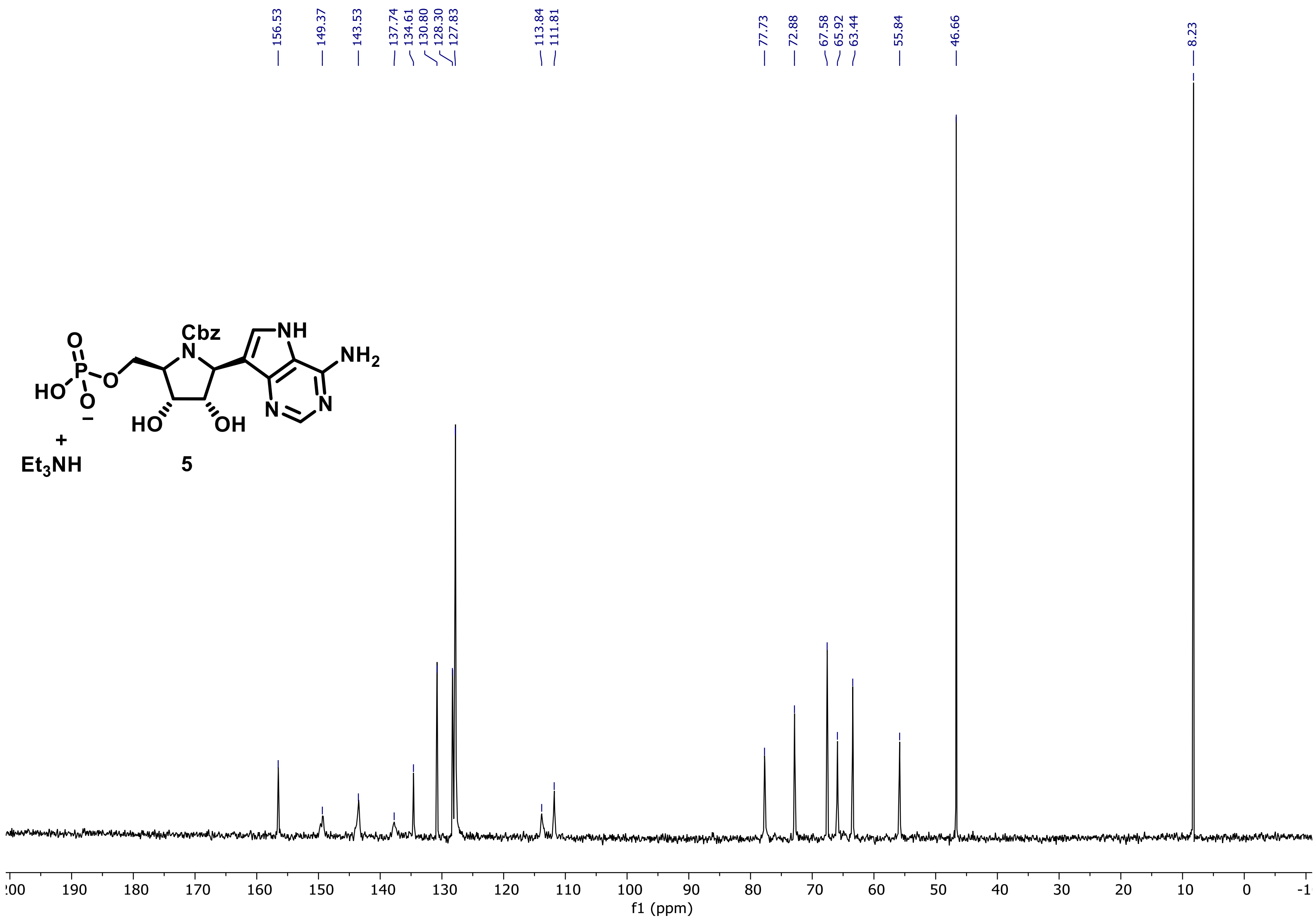

5

^{31}P NMR (202 MHz, D_2O)

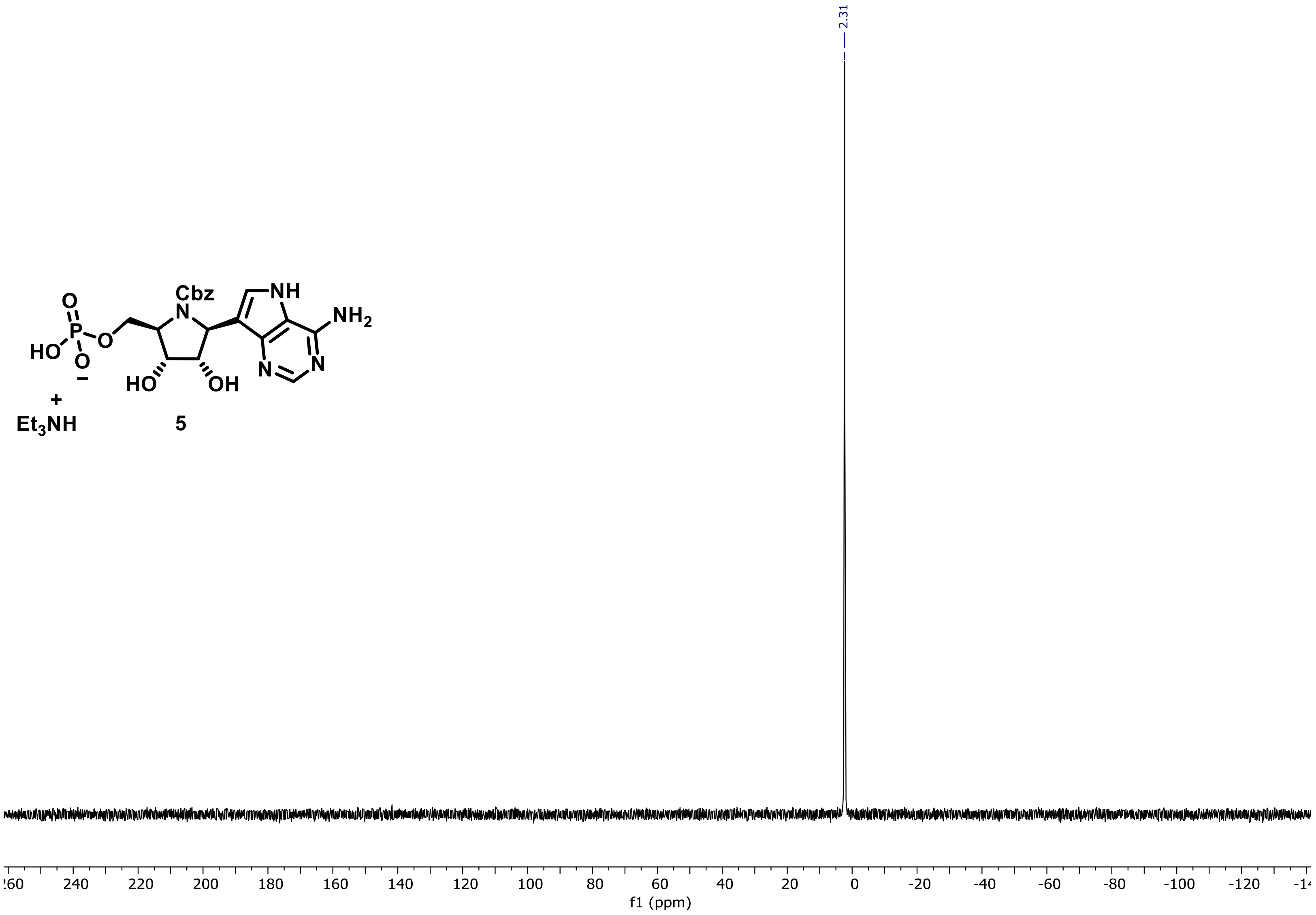

HRMS (ESI/QTOF)

Elemental Composition Report

Page 1

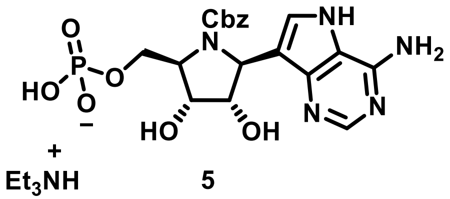
5

Single Mass Analysis

Tolerance = 5.0 mDa / DBE: min = -1.5, max = 150.0
Element prediction: Off
Number of isotope peaks used for i-FIT = 3

Monoisotopic Mass, Even Electron Ions
164 formula(e) evaluated with 3 results within limits (up to 100 closest results for each mass)
Elements Used:
C: 0-120 H: 0-120 N: 5-5 O: 0-40 Na: 0-1 P: 1-1

RSh-AV20-072b- 9 (0.117) Cm (9:11)

10-Aug-2020
1: TOF MS ES-
1.44e+007

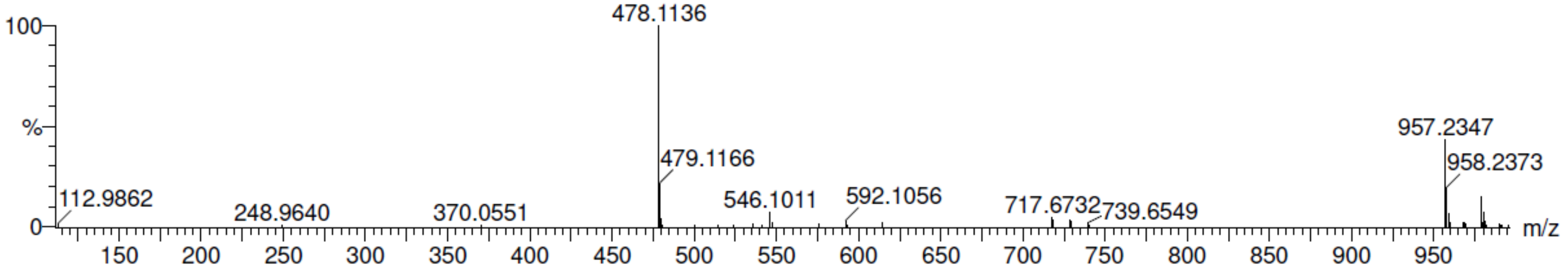

Minimum:				-1.5				
Maximum:		5.0	5.0	150.0				

Mass	Calc. Mass	mDa	PPM	DBE	i-FIT	Norm	Conf(%)	Formula
478.1136	478.1128	0.8	1.7	12.5	101.9	5.016	0.66	C19 H21 N5 O8 P
	478.1162	-2.6	-5.4	0.5	109.0	12.127	0.00	C10 H26 N5 O13 Na P
	478.1104	3.2	6.7	9.5	96.9	0.007	99.34	C17 H22 N5 O8 Na P

Compound **2**

^{1}H NMR (500 MHz, D_2O)

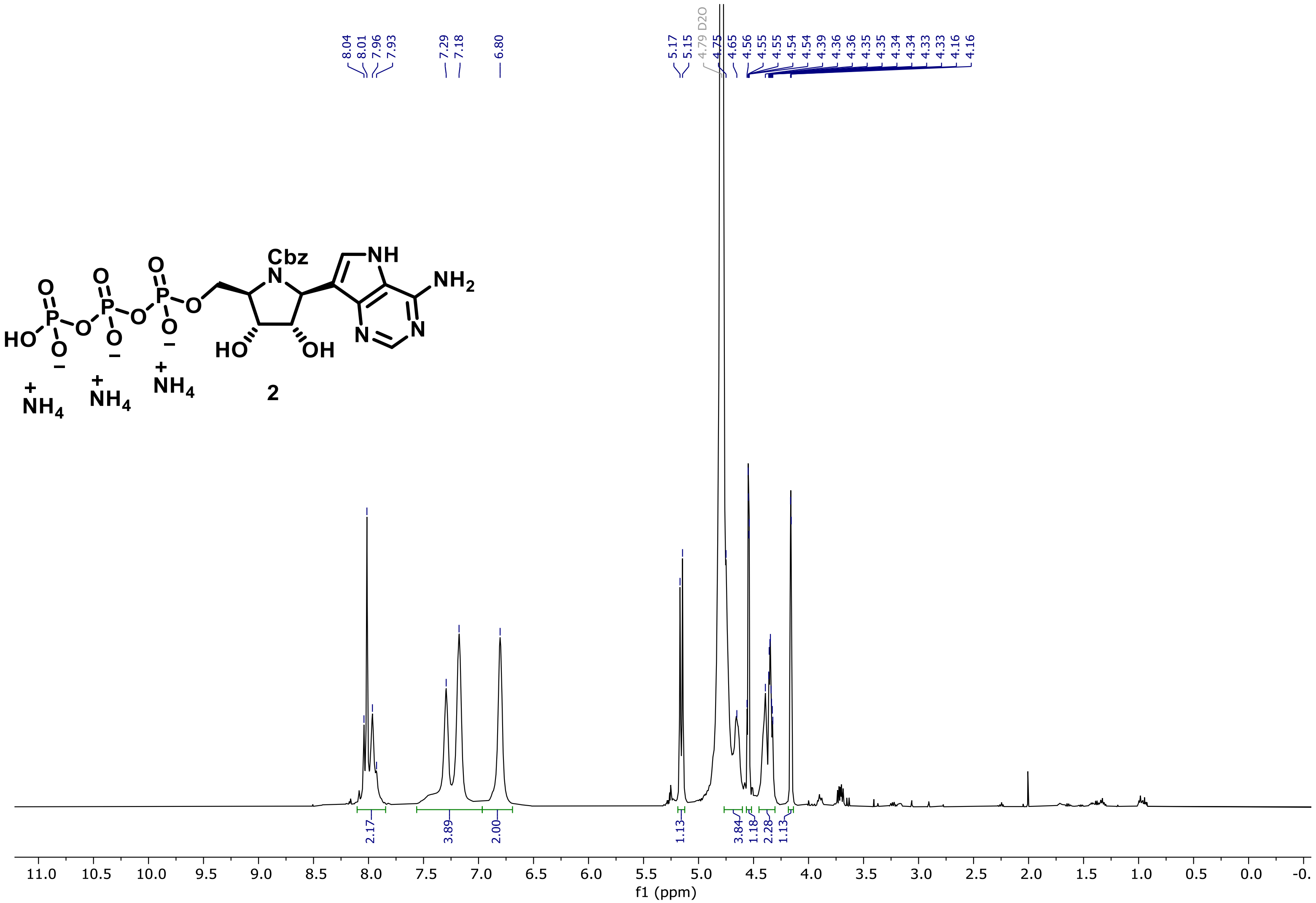

2

^{13}C NMR (126 MHz, D_2O)

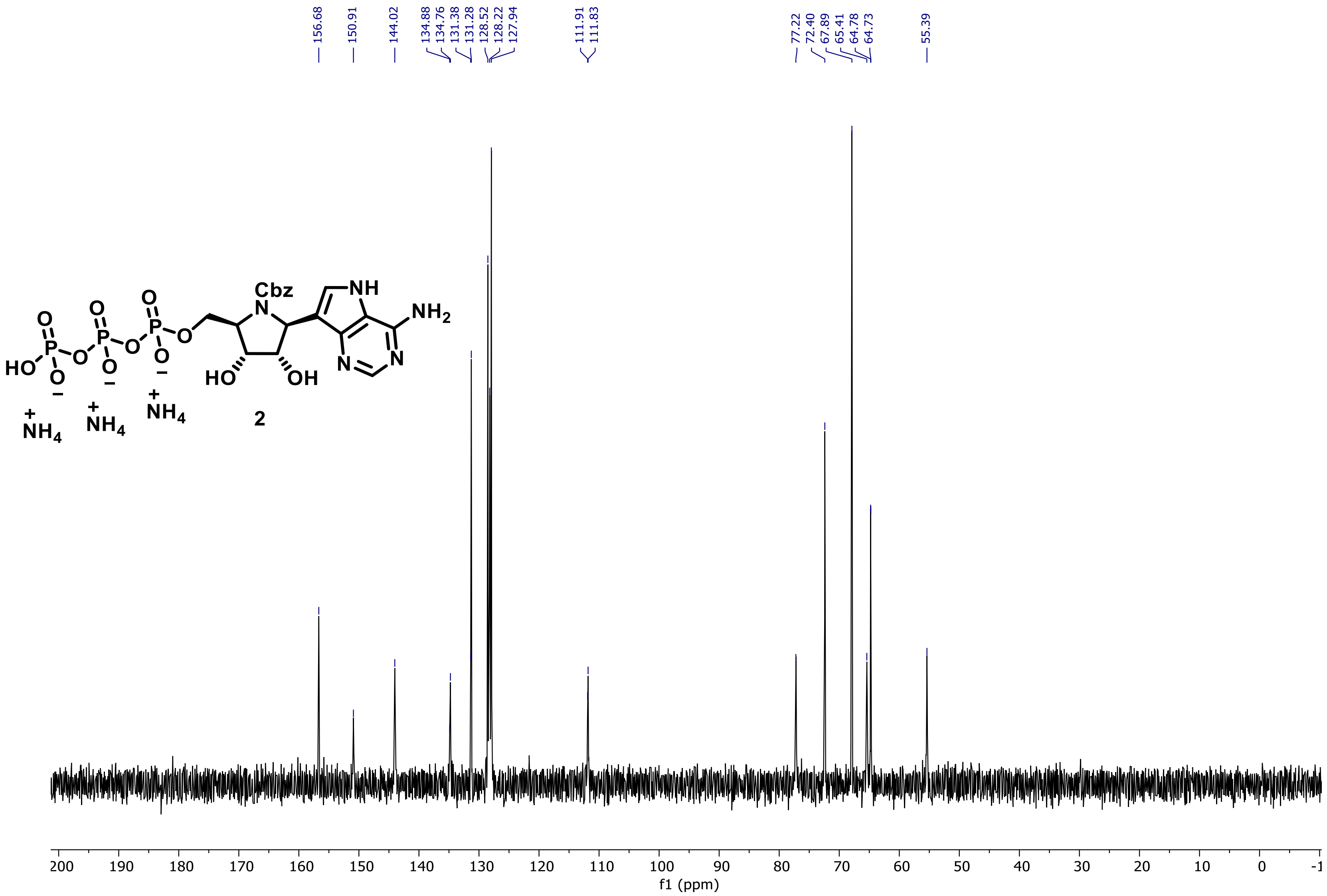

^{31}P NMR (202 MHz, D_2O)

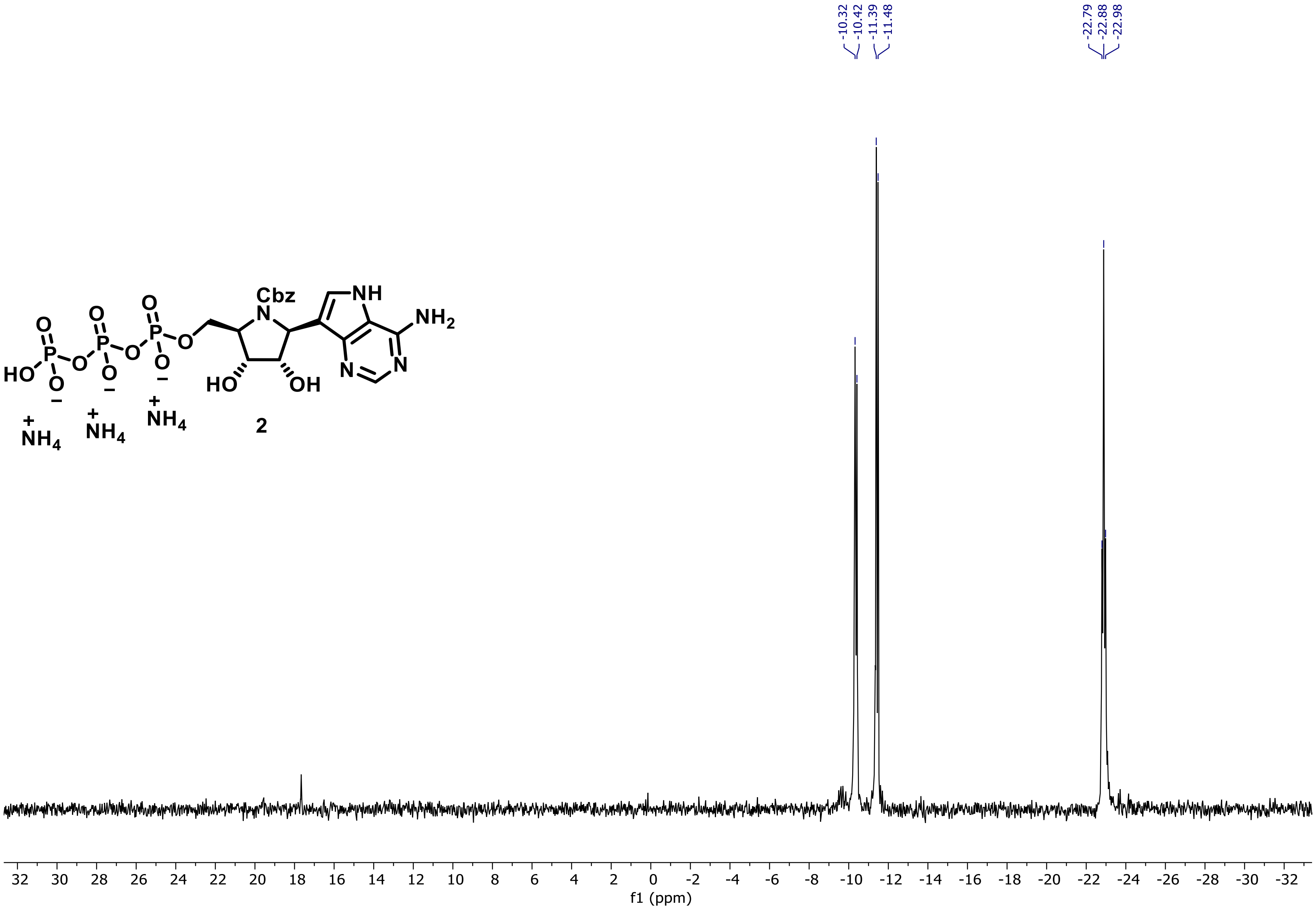

2

HRMS (ESI/QTOF)

Elemental Composition Report

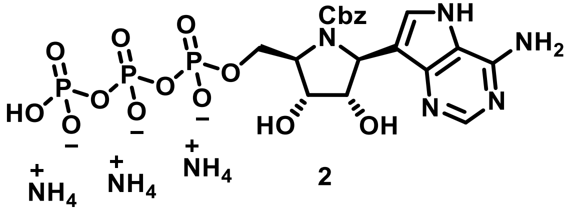

2

Single Mass Analysis

Tolerance = 5.0 mDa / DBE: min = -1.5, max = 150.0
Element prediction: Off
Number of isotope peaks used for i-FIT = 3

Monoisotopic Mass, Even Electron Ions
251 formula(e) evaluated with 6 results within limits (up to 100 closest results for each mass)
Elements Used:
C: 0-120 H: 0-150 N: 5-5 O: 0-40 Na: 0-1 P: 3-3

RSh-AV20-077c 14 (0.160) Cm (8:22-1:6)

19-Aug-2020
1: TOF MS ES+
1.31e+008

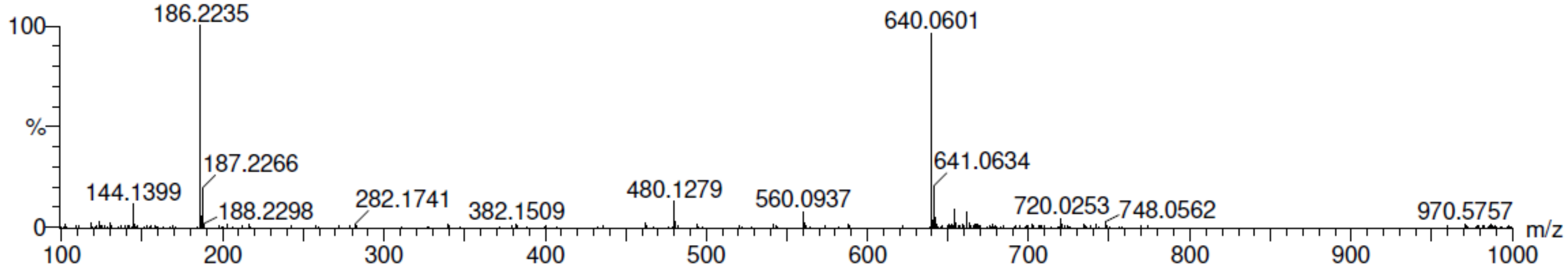

Mass	Calc. Mass	mDa	PPM	DBE	i-FIT	Norm	Conf(%)	Formula
Minimum:				-1.5				
Maximum:		5.0	5.0	150.0				
640.0601	640.0611	-1.0	-1.6	11.5	144.9	7.249	0.07	C19 H25 N5 O14 P3
	640.0587	1.4	2.2	8.5	137.7	0.001	99.93	C17 H26 N5 O14 Na P3
	640.0622	-2.1	-3.3	30.5	154.8	17.106	0.00	C35 H18 N5 O Na P3
	640.0646	-4.5	-7.0	33.5	156.1	18.372	0.00	C37 H17 N5 O P3
	640.0646	-4.5	-7.0	-0.5	150.8	13.076	0.00	C10 H30 N5 O19 Na P3
	640.0552	4.9	7.7	20.5	152.6	14.878	0.00	C26 H21 N5 O9 P3

Galidesivir triphosphate

^1H NMR (500 MHz, D_2O)

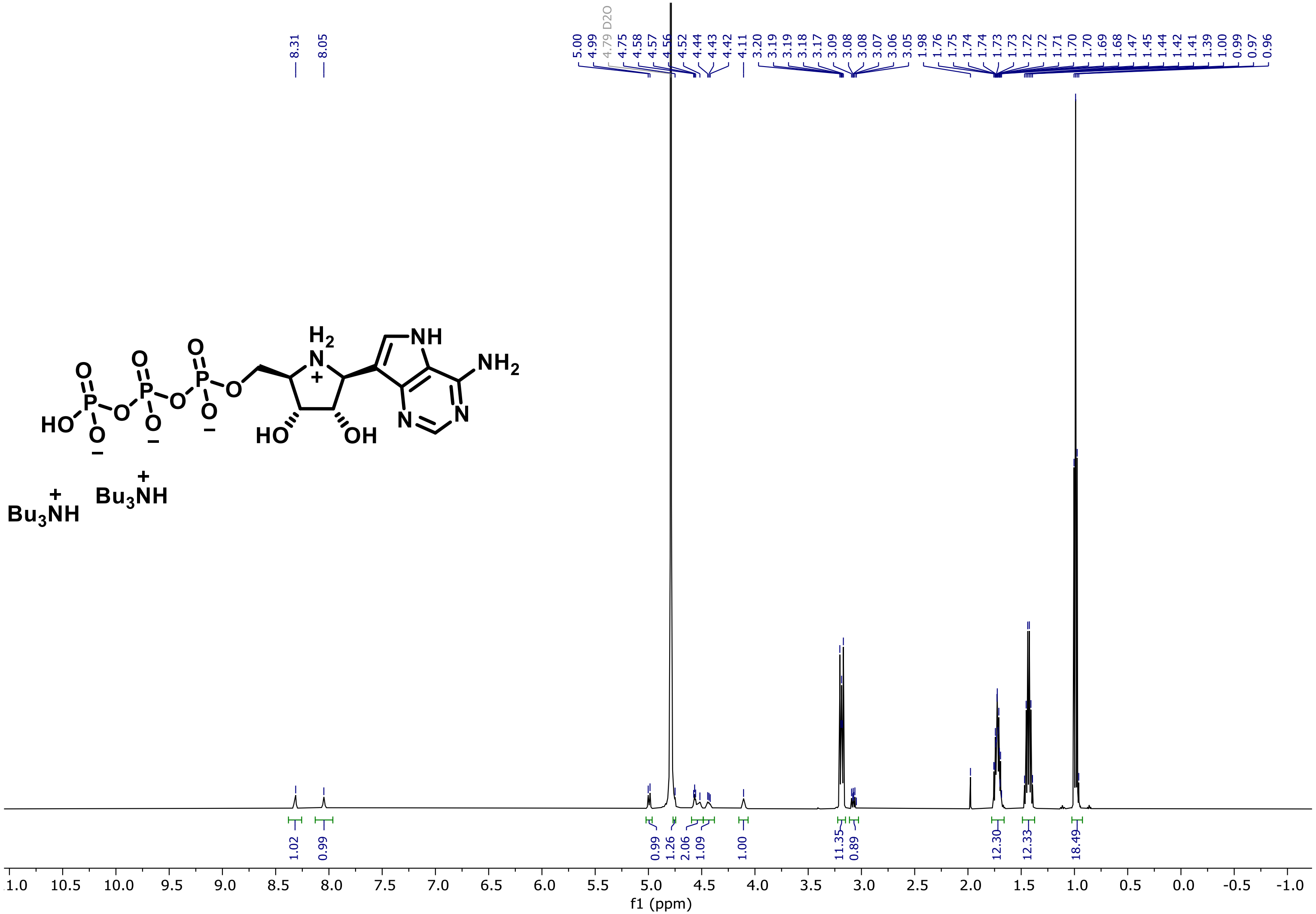

^{13}C NMR (126 MHz, D_2O)

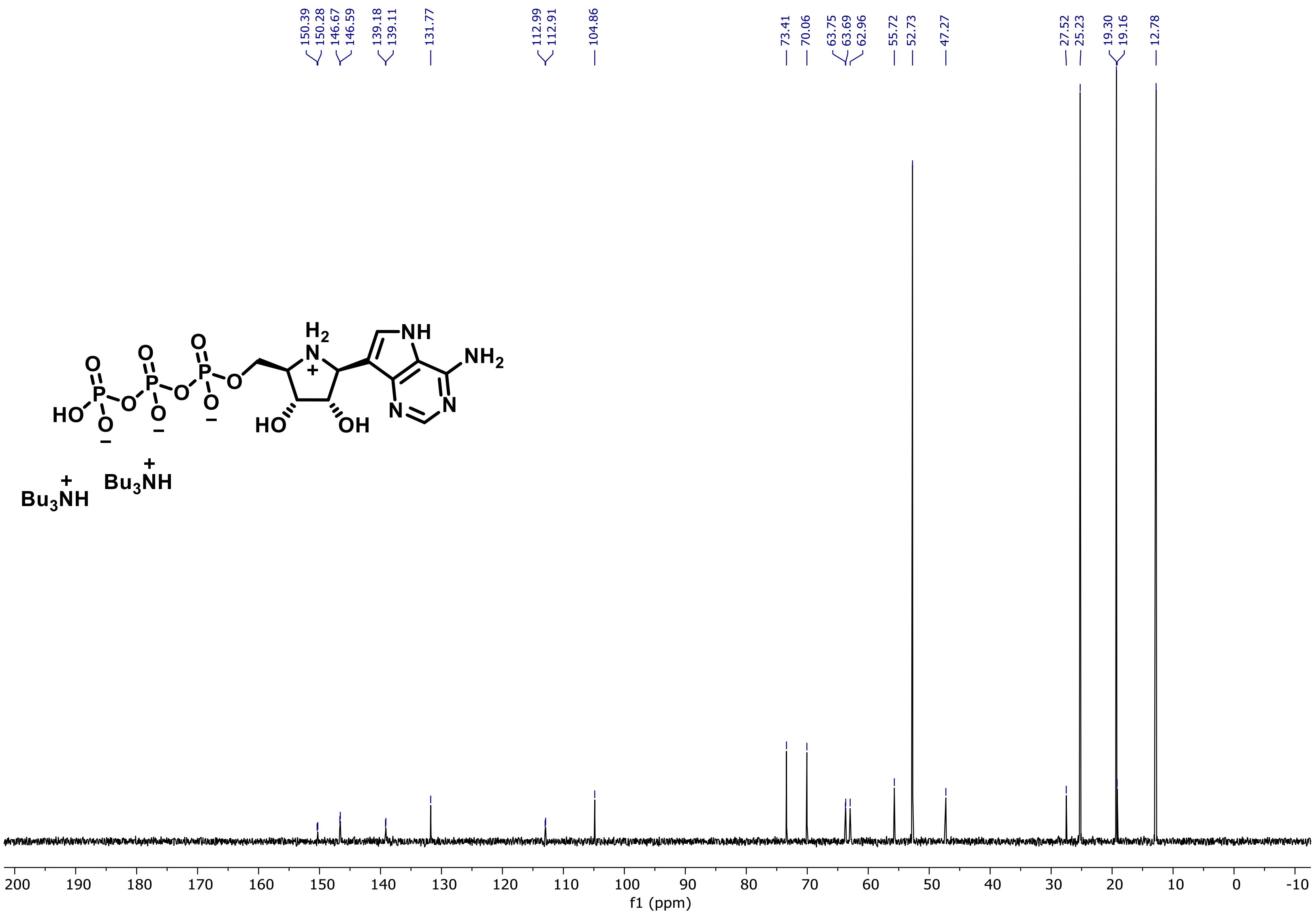

^{31}P NMR (202 MHz, D_2O)

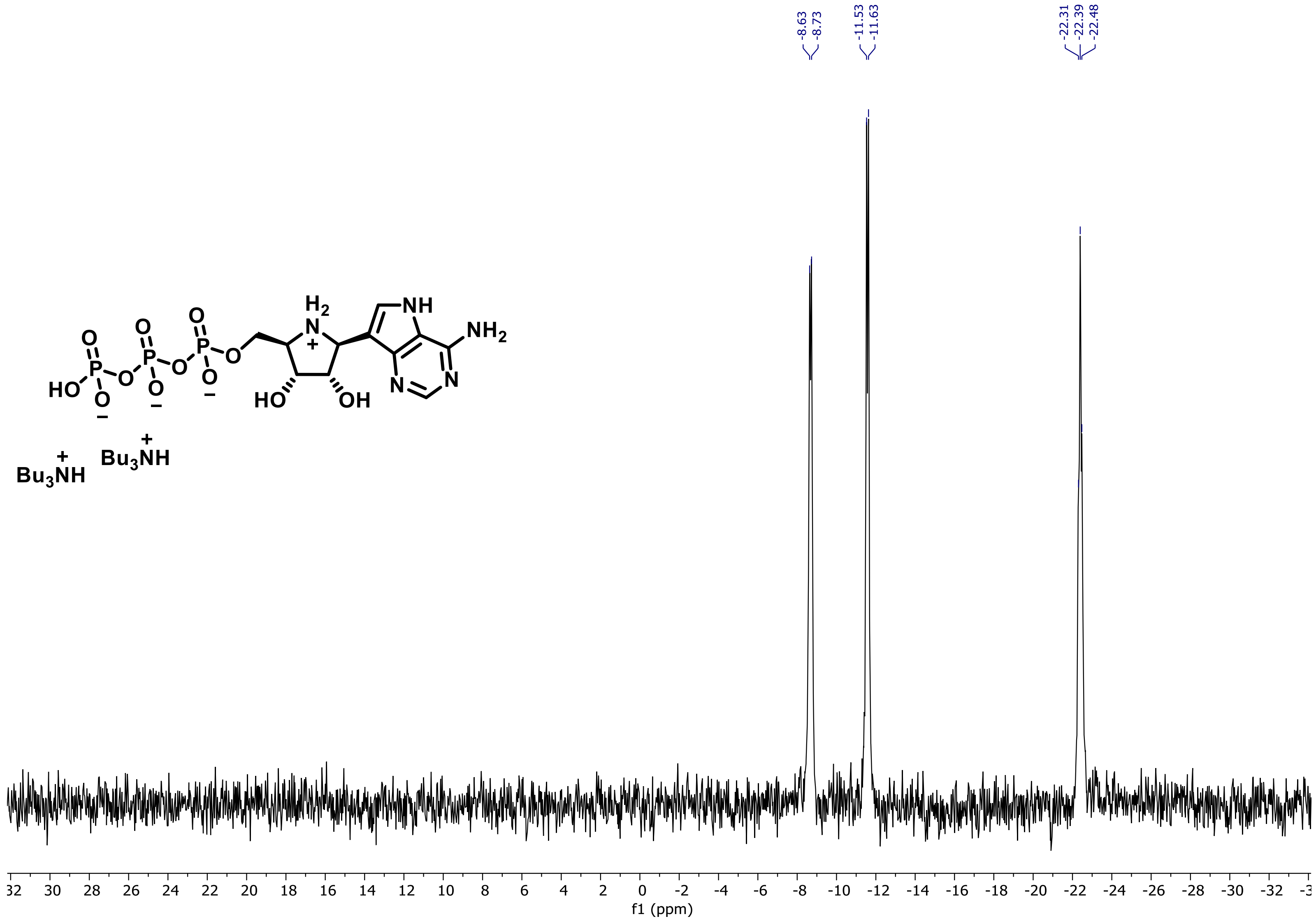

HRMS (ESI/QTOF)

Elemental Composition Report

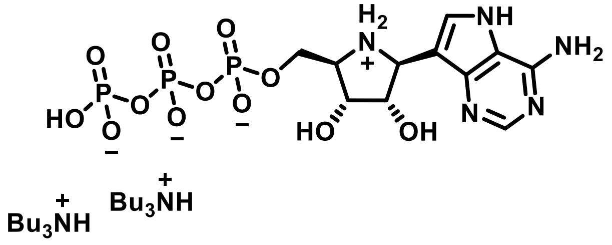

Single Mass Analysis

Tolerance = 5.0 mDa / DBE: min = -1.5, max = 150.0
Element prediction: Off
Number of isotope peaks used for i-FIT = 3

Monoisotopic Mass, Even Electron Ions
145 formula(e) evaluated with 2 results within limits (up to 100 closest results for each mass)
Elements Used:
C: 0-120 H: 0-150 N: 5-5 O: 0-40 Na: 0-1 P: 3-3

RSh-AV20-080b- 12 (0.143) Cm (9:19-1:7)

25-Aug-2020
1: TOF MS ES-
1.30e+008

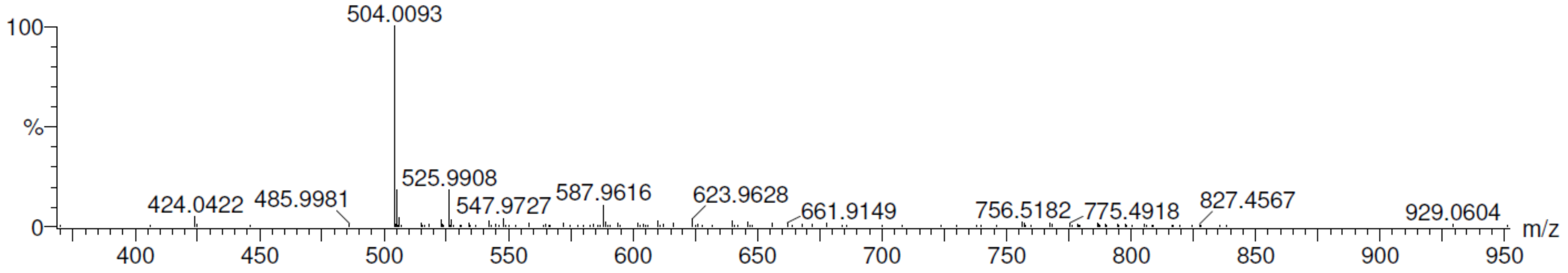

Minimum:				-1.5				
Maximum:		5.0	5.0	150.0				
Mass	Calc. Mass	mDa	PPM	DBE	i-FIT	Norm	Conf(%)	Formula
504.0093	504.0087	0.6	1.2	7.5	187.9	0.069	93.37	C11 H17 N5 O12 P3
	504.0063	3.0	6.0	4.5	190.6	2.713	6.63	C9 H18 N5 O12 Na P3

LCMS

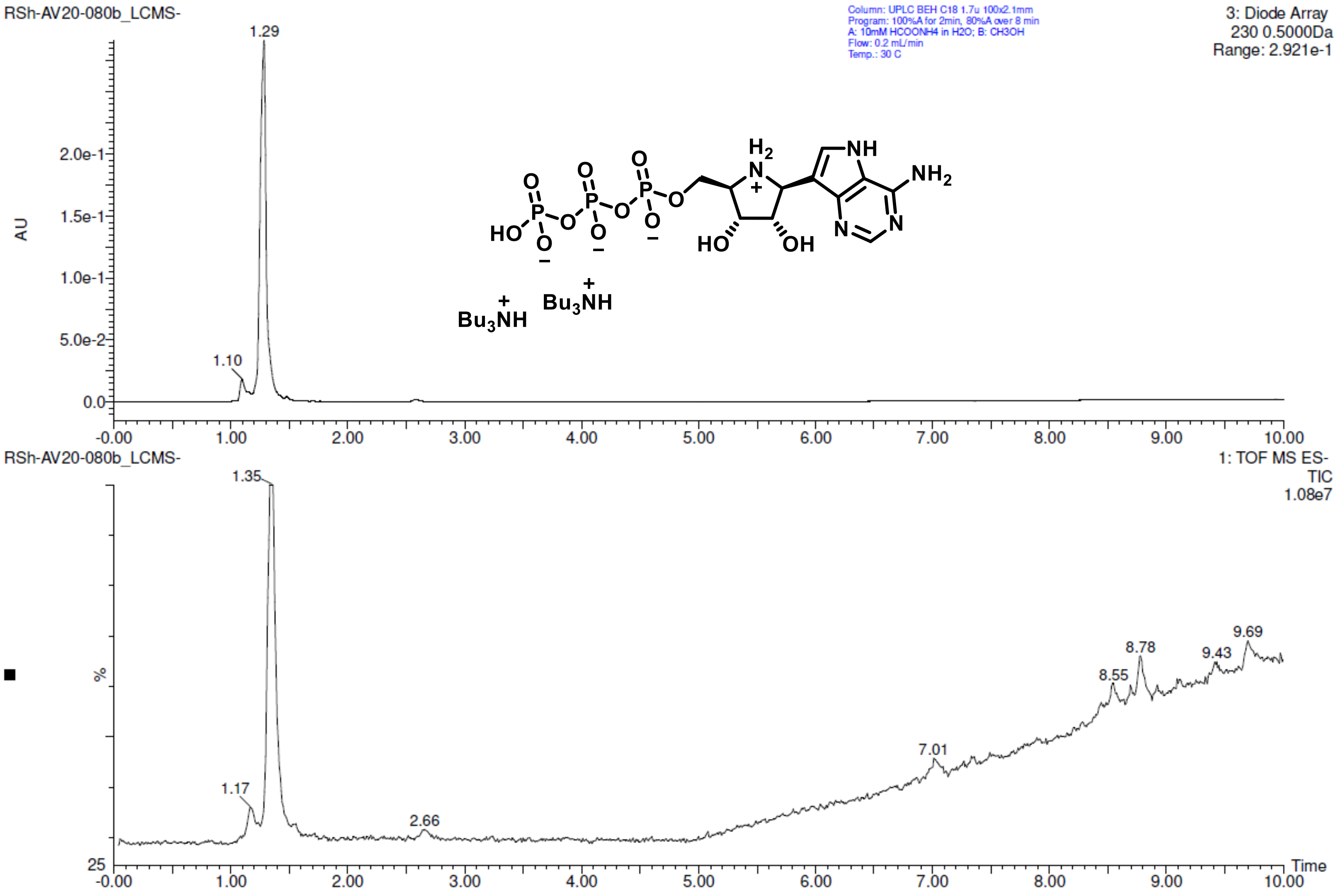
RSh-AV20-080b_LCMS-
Column: UPLC BEH C18 1.7u 100x2.1mm
Program: 100%A for 2min, 80%A over 8 min
A: 10mM HCOONH4 in H2O; B: CH3OH
Flow: 0.2 mL/min
Temp.: 30 C
3: Diode Array
230 0.5000Da
Range: 2.921e-1
1.29
1.10
AU
2.0e-1
1.5e-1
1.0e-1
5.0e-2
0.0
-0.00
1.00
2.00
3.00
4.00
5.00
6.00
7.00
8.00
9.00
10.00
RSh-AV20-080b_LCMS-
1: TOF MS ES-
TIC
1.08e7
1.35
1.17
2.66
7.01
8.55
8.78
9.43
9.69
%
25
Time
